# Cavity Quantum Electrodynamics Enables *para*- and *ortho*-Selective Electrophilic Bromination of Nitrobenzene

**DOI:** 10.1021/jacs.4c04045

**Published:** 2024-05-30

**Authors:** Braden M. Weight, Daniel J. Weix, Zachary J. Tonzetich, Todd D. Krauss, Pengfei Huo

**Affiliations:** † Department of Physics and Astronomy, University of Rochester, Rochester, New York 14627, United States; ‡ Department of Chemistry, University of Wisconsin-Madison, Madison, Wisconsin 53706, United States; ¶ Department of Chemistry, University of Texas at San Antonio, San Antonio, Texas 78249, United States; § Department of Chemistry, University of Rochester, Rochester, New York 14627, United States; ∥ The Institute of Optics, Hajim School of Engineering, University of Rochester, Rochester, New York 14627, United States

## Abstract

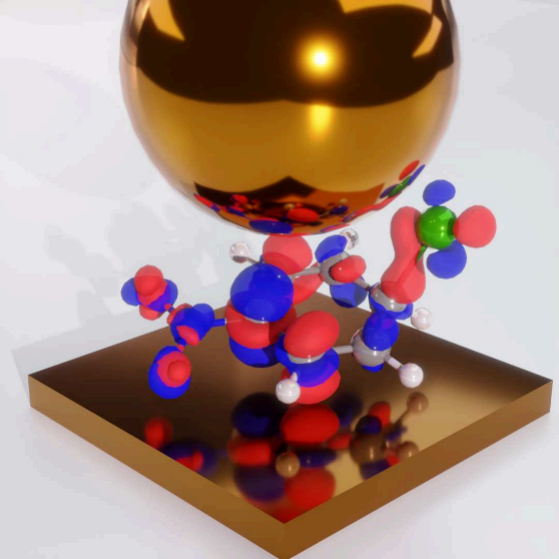

Coupling molecules to a quantized radiation field inside an optical cavity has shown great promise to modify chemical reactivity. In this work, we show that the ground-state selectivity of the electrophilic bromination of nitrobenzene can be fundamentally changed by strongly coupling the reaction to the cavity, generating *ortho*- or *para*-substituted products instead of the *meta* product. Importantly, these are products that are not obtained from the same reaction outside the cavity. A recently developed *ab initio* approach was used to theoretically compute the relative energies of the cationic Wheland intermediates, which indicate the kinetically preferred bromination site for all products. Performing an analysis of the ground-state electron density for the Wheland intermediates inside and outside the cavity, we demonstrate how strong coupling induces reorganization of the molecular charge distribution, which in turn leads to different bromination sites directly dependent on the cavity conditions. Overall, the results presented here can be used to understand cavity induced changes to ground-state chemical reactivity from a mechanistic perspective as well as to directly connect frontier theoretical simulations to state-of-the-art, but realistic, experimental cavity conditions.

## Introduction

Coupling molecules to a quantized radiation field inside an optical cavity creates a set of photon–matter hybrid states called polaritons. These polariton states hold great promise for changing chemical reactivity in a general and facile way by tuning the properties of matter as well as the properties of photons. Despite the theoretical predictions of using polaritons for novel chemistry broadly,^1^ what has been demonstrated experimentally largely relates to polariton-modified reaction kinetics. For example, collective couplings between the electronic excited states of fulgide or similar molecules and quantized photonic modes inside an optical cavity, so-called electronic strong coupling (ESC), were shown to both enhance or suppress photochemical isomerization reactions.^2,3^ In another example, vibrational excitations collectively coupled to the photonic excitations of a microcavity, commonly referred to as vibrational strong coupling (VSC), resulted in chemical kinetics that can be enhanced^4,5^ or suppressed.^6−8^ In these two collective coupling regimes, the kinetics of the reactions are changed, but importantly, there is no new type of product generated compared to the same reactions outside the cavity.

Recent theoretical investigations^1,9^ have suggested that the ground state of a molecular system can be significantly modified by coupling the electronic states of a molecule to a cavity photon mode.^10−20^ In particular, it has been shown that the cavity can modify the endo/exo selectivity of Diels–Alder reactions,^21,22^ modify the ground-state proton transfer reaction barriers and driving forces,^15,16^ and selectively control the product of a click reaction.^23^ Note that the cavity frequency in these studies is chosen to be in the range of electronic excitations in molecules (in terms of energy, on the order of eV), and thus, the resulting polaritonic effects are expected to be different than the more commonly explored VSC regime.^6,8^

In addition, for this case cavity modified ground-state chemistry does not require the usual resonance effects of light–matter interactions (i.e., frequency matching between light and matter excitations) since the cavity can directly modify how the ground state of the hybrid system couples to the molecular system through the cavity mode’s vacuum fluctuations, referred to as quantum vacuum fluctuation modified chemistry.^15,21^ Importantly, predictions based on single molecules coupled to a cavity are also within the reach of the magnitude of the strong coupling shown in recent experiments using a plasmonic nanocavity.^24^ A conceptual understanding of these recently proposed ground-state modifications due to cavity vacuum fluctuations^9,15,17,21,25−27^ is provided in the Theoretical Methods section. From an experimental perspective, cavity vacuum fluctuations have already been shown to modify the work function of materials inside the cavity,^28^ as predicted by early theory work.^20^

The molecule–cavity hybrid system can be described by the Hamiltonian^1,9,29^ in eq 2. The light–matter coupling strength is expressed as
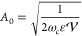
1where  is the effective mode volume of the cavity, ε is the permittivity inside the cavity, and ω_c_ is the cavity frequency. Through light–matter interactions, various photon-dressed electronic states will be coupled to each other. For example, the ground electronic state with 1 photon in the cavity and an excited electronic state with 0 photons in the cavity will couple through the light–matter interaction. When the energy of these two basis states become close, they interact (mathematically similar to the interaction of atomic orbitals to form molecular orbitals), leading to the formation of excited polariton states. These (and all similar) interactions represent the result of resonant, strong light–matter coupling, causing the formation of new eigenstates, i.e., polaritons.

Through nonresonant light–matter interactions, the cavity can directly modify the ground state of a molecule coupled to the cavity. In order to understand this effect, which is important for understanding how cavities can modify ground-state chemical reactivity, one has to go beyond the predictions of the simple Jaynes–Cummings model for light–matter interactions.^30^ Direct modification of polariton ground states can be caused by two physical processes:^1,9,31^ (i) off-resonance light–matter interactions (third term in eq 2) through the ground-state permanent dipole and optical transition dipoles between the ground and excited states and (ii) a dipole self-energy (DSE) term. The detailed theoretical arguments are provided in the Theoretical Methods section below eq 2.

**Scheme 1:**
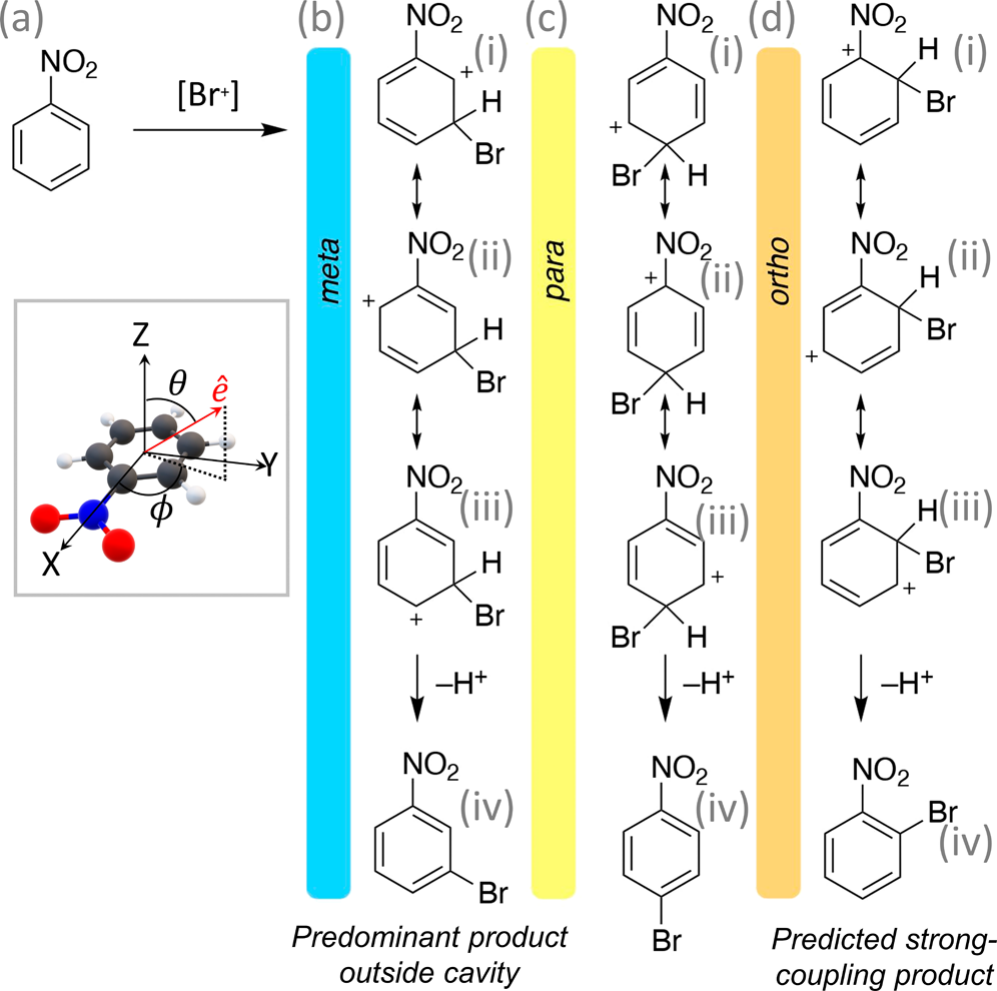
Mechanism and Regiochemistry for the Bromination of Nitrobenzene This reaction generally results in *meta*-substituted nitrobenzene (blue) with small or no detectable amounts of *ortho*- and *para*-products. The depicted mechanism, via a cationic intermediate, is generally used to explain this selectivity. Coupling this reaction to the cavity is predicted to change selectivity to favor the *para* (yellow) or *ortho* (orange) products.

In this work, we demonstrate that the strong coupling between molecule and cavity can dramatically change the reaction outcome of the bromination of nitrobenzene in its ground state. The bromination of nitrobenzene is a textbook electrophilic aromatic substitution (EAS) reaction,^32,33^ where *meta* bromination is the predominant product under many standard conditions.^34−39^ The observation of exclusively *meta* bromination has been well-explained by the relative stability of the three possible cationic intermediates of BrC_6_H_4_NO_2_^+^ (see Scheme 1) using resonance structure analysis.^33,40,41^ These and similar reactions have also been explored with density functional theory; although in some cases alternative mechanisms have been proposed,^42−46^ the Wheland intermediate explains the regiochemistry for nitrobenzene bromination in polar solvents well.

We apply a recently developed *ab initio* polariton chemistry approach^16^ and theoretically demonstrate that coupling nitrobenzene to an optical cavity can fundamentally change the selectivity of the electrophilic bromination reaction of nitrobenzene, making *ortho*- or *para*-substituted products possible. We emphasize that these modifications are achieved for highly off-resonant cavities which generate products inside the cavity that cannot be generated under the same conditions outside the cavity. We have further provided an analysis of how coupling to the cavity will change the charge distribution of the cationic intermediate, which therefore causes a modification to the preferred bromination site. As such, strong couplings between molecules inside the cavity offer a promising tool^14^ to fundamentally change the outcome of known chemical reactions.

## Results and Discussion

Scheme 1 presents the classic reaction mechanism of the electrophilic bromination of nitrobenzene, which first proceeds through a cationic intermediate (the so-called Wheland intermediate) PhNO_2_–Br^+^ that undergoes subsequent deprotonation to afford the product. In this work, we focus on the cavity modification of the energies of these positively charged reaction intermediates PhNO_2_–Br^+^. This molecule is accepted as the quasi-stable intermediate species in the kinetics of the bromination of nitrobenzene, and the site selectivity of halogenations of aryl species is largely dictated by this intermediate.^32,40,41,47^ However, recent experimental and theoretical explorations have demonstrated that other pathways, such as an addition–elimination route, maybe more favored under certain conditions.^42,48,49^

Outside the cavity, the *meta* intermediate is the most stable for nitrobenzene and provides nearly 100% selectivity due to a favorable set of possible resonance structures. On the other hand, the *para*- and *ortho*-substituted products are not observed due to the presence of high-energy resonance structures. In the case of bromination outside of the cavity, which corresponds to the parameters *A*_0_ = 0.0 a.u. and ω_c_ = 0.0 eV in eq 2, the *meta*-substituted intermediate species is more thermodynamically stable compared to the *ortho* and *para* species by roughly 2 and 5 kcal/mol, respectively, confirming this classic reaction mechanism.

**Figure 1:**
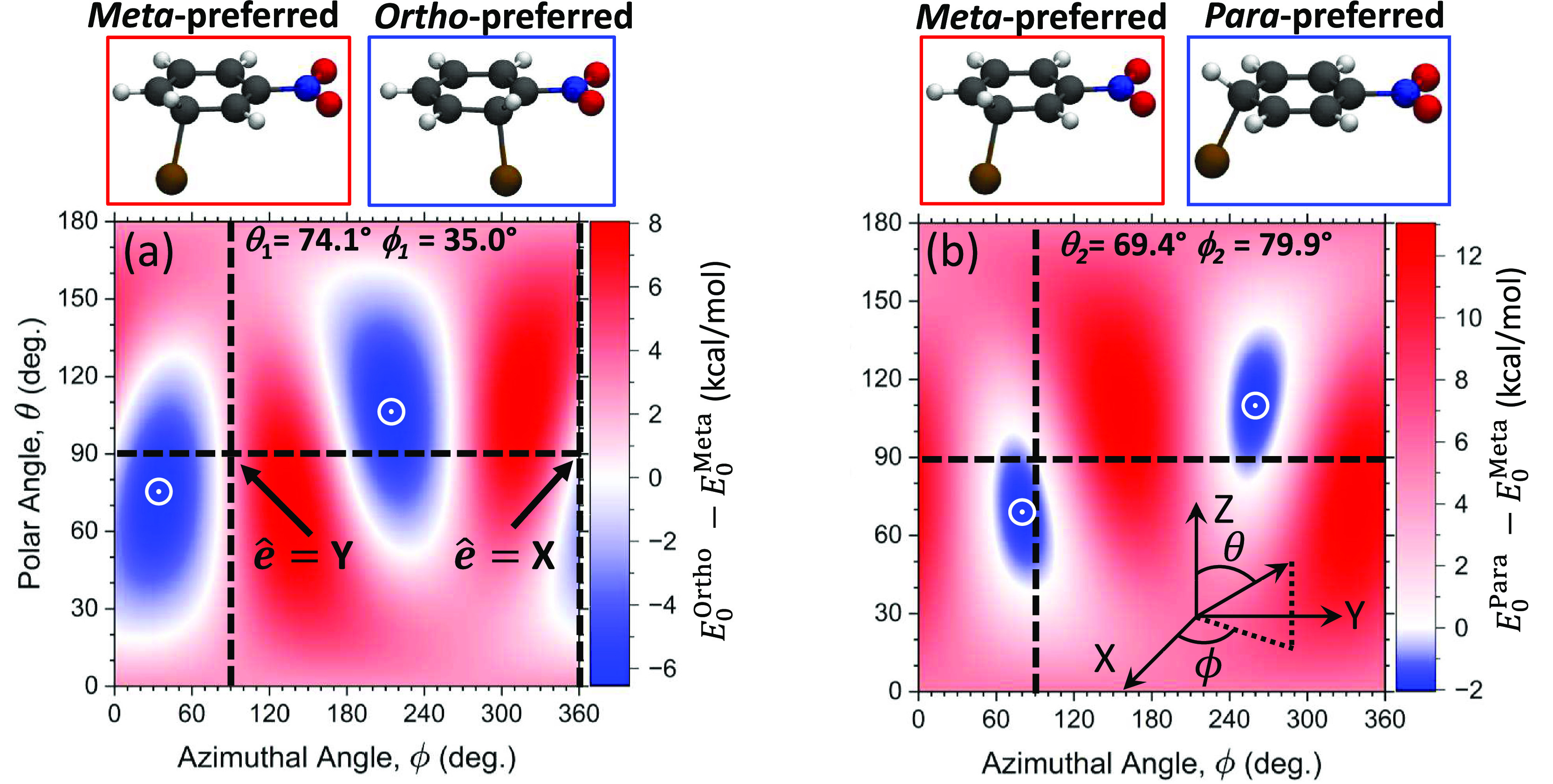
Relative energy of the polaritonic ground states between (a) *ortho*-BrC_6_H_4_NO_2_^+^ and *meta*-BrC_6_H_4_NO_2_^+^ and between (b) *para*-BrC_6_H_4_NO_2_^+^ and *meta*-BrC_6_H_4_NO_2_^+^ as a function of the azimuthal ϕ ∈ [0, 2π) and polar θ ∈ [0, π) angles of the cavity polarization vector with respect to the molecular Cartesian axes in the inset of panel (b). The cavity frequency and coupling strength are fixed at ω_c_ = 1.8 eV and *A*_0_ = 0.3 a.u. (corresponding to a cavity volume of  nm^3^ or a field strength of  V/nm). The minimum values of the relative energies in each case are (a) −6.43 and (b) −1.97 kcal/mol, which are denoted by white open circles.

Coupling this intermediate with the optical cavity, we find that the energy of the *ortho*- and *para*-substituted intermediates can be lower than that of the *meta*-substituted species under a range of coupling strengths *A*_0_ and cavity frequencies ω_c_. The relative energies of different intermediates depend on the orientation of the intermediate relative to the electric field of the cavity. A more stable *ortho*-substituted intermediate is formed if the cavity polarization is along one direction of the molecule (blue regions in Figure 1a; Cartesian directions are defined in the inset to Scheme 1a), while the *para* substituent becomes stabilized for a different polarization direction (blue regions in Figure 1b).

To observe a significant change in the polaritonic ground state (due to the nonresonant light–matter coupling terms in the Hamiltonian), it generally requires a very strong light–matter interaction strength between a single molecule and the cavity mode.^9,15,21^ This light–matter coupling regime can be realized by using state-of-the-art plasmonic nanocavities, which provide realistic experimental parameters that were used for these simulations. In these systems,^50,51^ the cavity mode volume is extremely small, on the order of Å^3^. For example, a recent nanocavity achieved a mode volume^52^ of  nm^3^. The coupling strength typically required^21^ to observe any interesting changes to the ground state is  a.u., corresponding to *A*_0_ ≈ 0.3 a.u. or a field strength of  V/nm that can be experimentally accomplished.^24^ Experimentally, the nanoparticle-on-mirror (NPoM) cavity structure^53^ can achieve such coupling strengths with electric field intensities  V/nm. For the specific case of a gold plasmonic nanocavity,^50,51^ the cavity frequency is ω_c_ = 1.8 eV. These coupling strengths from NPoM cavities are also consistent with a mode volume of  nm^3^.

Figure 1a presents the relative energetic stability of the *meta* and *ortho* cationic intermediate species, computed as  inside the cavity as a function of θ and ϕ. Figure 1b presents the results of . The cavity polarization direction  with respect to the **X**-, **Y**-, and **Z**-directions of the molecule is defined by the polar angle θ and the azimuthal angle ϕ (see inset to Scheme 1a and the inset of Figure 1b). In Figure 1, all possible spatial orientations of the cavity polarization direction  with respect to the molecule are presented. When θ = 90° and ϕ = 0° (or equivalently, ϕ = 360°), , and when θ = 90° and ϕ = 90°, . Note that in the experimental setup, the cavity polarization direction is fixed^50^ and is related to the cavity design, whereas the molecular orientation is expected to be random unless using additional geometrical confinement.^50^ We want to explore all possible relative orientations between the molecule and cavity polarization. To have a simple coordinate system, we fix the molecular orientation (equivalent to defining the coordinate system with respect to the molecule) and subsequently vary the cavity polarization direction with respect to the molecular orientation, which is equivalent to having a fixed cavity polarization and varying the molecular orientation.

From Figure 1, we can see the regions (blue) of θ and ϕ where *ortho*- or *para*-substituted intermediates are more stable than the *meta*-substituted isomer. For example, the most stable energy for the *ortho*-substituted complex is achieved when θ ≈ 75° and ϕ ≈ 35°, which is 6.4 kcal/mol lower in energy than the *meta*-substituted complex. The most stable energy for the *para*-substituted complex is achieved when θ ≈ 70° and ϕ ≈ 80°, which is 2.0 kcal/mol more stable compared to the *meta*-substituted complex. When θ = 0° for  or θ = 180° for , the cavity polarization is along **Z**-direction, where the molecular dipole is nearly zero and the cavity modification diminishes, as expected. The room-temperature thermal energy is *k*_B_*T* ≈ 0.58 kcal/mol, and for the case of bromination outside the cavity, the *meta*-substituted intermediate species is thermodynamically stable compared to the *ortho* and *para* species by roughly 2 and 5 kcal/mol, respectively (see Figure 4). Thus, these results suggest that by coupling the nitrobenzene molecule to an optical cavity, the preferred bromination sites can be tuned to either *ortho*-substituted or *para*-substituted, whereas outside the cavity, *meta*-substituted products dominate under standard conditions.^32,33^ As such, coupling to the cavity allows one to obtain “impossible products” (*para*- and *ortho*-substituted BrC_6_H_4_NO_2_^+^) outside the cavity. For additional comparison, Figure S10 shows the energy difference between the *ortho* and *para* species.

For the single molecule strong coupling case, one often has to control the molecular orientation with respect to the cavity field polarization direction in order to see polaritonic effects on the chemistry. For example, recent theoretical work suggests that only when coupling to the cavity along particular electric field directions can one selectively obtain the endo or exo products of a Diels–Alder reaction,^21^ whereas an isotropic random orientation of the molecule will likely end up giving an equal mixture of both isomers, a situation similar to that obtained outside the cavity. Experimentally, it has been shown for a single molecule–NPoM cavity system that controlling the molecular orientation to align the molecule with the cavity field is possible.^50^ Nevertheless, perfectly controlling the molecular orientation in all cases is exceptionally challenging.

The currently proposed bromination reaction, on the other hand, does not require precise control of the orientation of molecules, if the goal is to obtain non-*meta*-substituted products in order to demonstrate the use of a cavity to enable novel bromination selectivity under standard reaction conditions. As such, randomly orientated molecules strongly coupled to the nanocavity will bias the selectivity to favor *ortho*- and *para*-substituted nitrobenzene products. On the other hand, if the objective is to only obtain either *ortho*- or *para*-substituted pure species, then one would need to either control the molecular orientation along the cavity field polarization or separate the mixture of products post-reaction, as is common for bromination of activated arenes.

To further understand the role of the optical cavity in inducing these chemical changes, we compute the ground-state electron density difference between molecules placed inside the cavity and outside the cavity. This comparison allows for a direct visualization of the cavity mediated changes to the electron density and facilitates chemical insights into the relative stability of the various substituted reaction intermediates. The difference density function of the ground state is defined as , where  is the polaritonic ground-state electronic density (with the photonic degrees of freedom integrated out), and ξ_00_ is the bare electronic ground-state density. Theoretical details for computing  and ξ_00_(*X*, *Y*, *Z*) are provided in the Supporting Information. Further, a discussion regarding the contributions to the polaritonic ground-state density matrix with respect to the calculated the electronic densities can be found in the Supporting Information. To help visualize the density difference, we further integrate out the *Z*-direction (perpendicular to the plane of the benzene ring) and present the two-dimensional density differences, Δρ(*X*, *Y*) = ∫ d*Z*Δρ(*X*, *Y*, *Z*).

**Figure 2:**
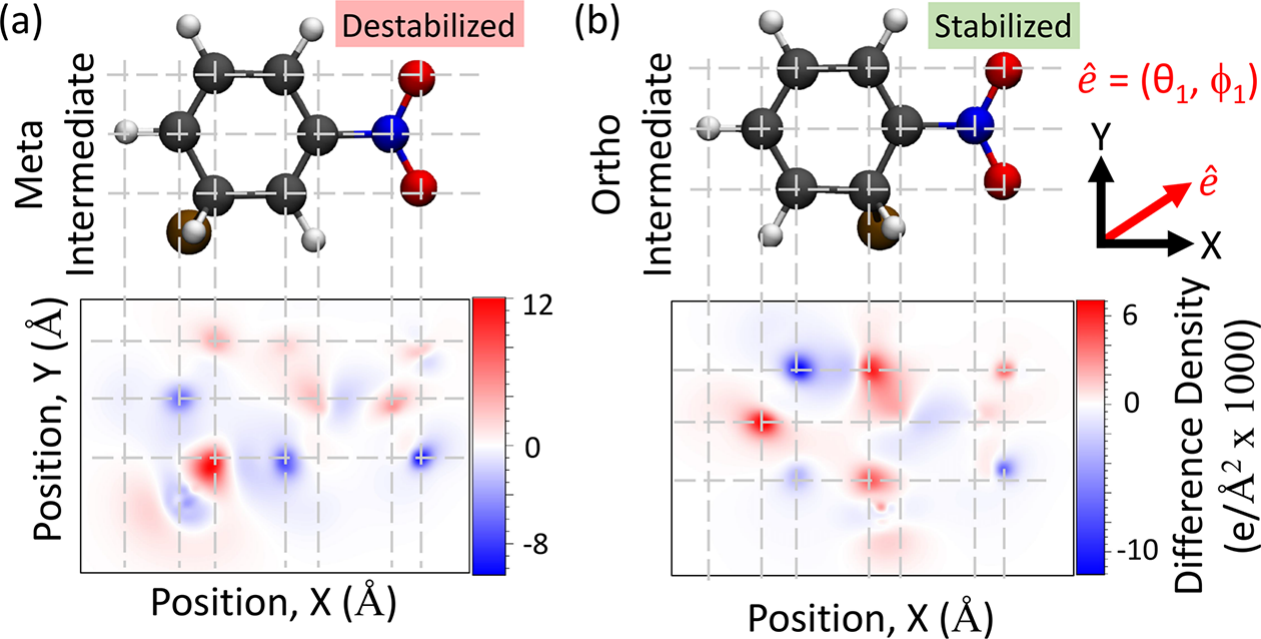
Ground-state density difference  of (a) the *meta* cationic intermediate and (b) the *ortho* cationic intermediate with a light–matter coupling strength of *A*_0_ = 0.3 a.u. and cavity frequency ω_c_ = 1.8 eV. The cavity polarization is along , defined in the inset of Figure 1b, showing the projection of the polarization onto the *XY*-plane. The color bar indicates the magnitude and sign of the difference density Δρ_00_(*x*, *y*), where positive (red) indicates electron charge accumulation and negative (blue) indicates electron charge depletion upon coupling the molecule with the cavity.

Figure 2 shows the electron density difference when the cavity polarization is along  (see Figure 1a and the inset of Figure 2b for the polarization direction projected onto the *XY*-plane). The rest of the parameters are the same as in Figure 1, with *A*_0_ = 0.3 a.u. and ω_c_ = 1.8 eV. Under these conditions, the *ortho*-substituted Wheland intermediate becomes more stable than the *meta*-substituted intermediate (see Figure 1a) by ∼6.4 kcal/mol. Figure 2a shows the density difference for the *meta*-substituted reaction intermediate, while Figure 2b presents the density difference for the *ortho*-substituted reaction intermediate. The color scheme of this plot is as follows: red (positive values) indicates the accumulation of electron density, and blue (negative values) indicates the depletion of electron density upon coupling of the molecule into the cavity. Figure S3 in the Supporting Information shows data for the *meta*- (Figure S3a) and *para*-substituted (Figure S3b) intermediate species.

Figure 2 presents the electron density difference contour maps for the *meta*-substituted and *ortho*-substituted intermediate species, depicting the cavity induced localization of the electron density accumulation (red) and depletion (blue). For the *meta*-substituted intermediate species, the bromine-connected carbon accumulates a large amount of electron density when coupling to the cavity, while the adjacent carbons (*ortho* and *para* carbons relative to the nitro group) exhibit strong electron density depletion. On the other hand, the *ortho* intermediate species in Figure 2b shows a delocalized electron density accumulation on the bromine-connected carbon and the two carbons in the *meta* position relative to the bromine-connected carbon. However, the electron density becomes depleted only at the *para* carbon relative to the bromine-connected carbon (i.e., the carbon opposite the bromine-connected carbon).

This reorganization of the electron density allows for the cavity mediated selectivity of the three cationic intermediate species. In fact, for the *ortho* intermediate species, one can make a direct connection to a resonance structure for the Wheland intermediate that contains a partial positive charge on the carbon opposite the bromine-connected carbon. Thus, the cavity stabilizes the *ortho* Wheland intermediate by shifting the electronic density to a stable resonance structure, depicted in Scheme 1d(ii), in contrast to the destabilizing resonance structure that occurs outside the cavity with a partial positive charge in the nitrogen-connected carbon, depicted in Scheme 1d(i). In other work, the reorganization of the ground-state electronic distribution inside the cavity has been theoretically observed^11,14,22,54^ and attributed to the exchange of character between molecular orbitals.^14,22^ This effect is highly system-dependent since the observed effects depend on the relative orientation and strengths of the permanent and transition dipole moments of the molecular orbitals as well as their squares through the DSE. While a general theory for how the electronic density changes inside the cavity remains unknown, it will be the subject of future work to figure out design principles for at least a given class of reactions. On the other hand, in the collective VSC regime (when ω_c_ ≈ 0.1 eV), recent experiments^55^ show that the nuclear magnetic resonance (NMR) spectrum of molecules is not modified inside the cavity (i.e., there are no apparent NMR shifts under VSC), implying that the electronic density is not perturbed by the cavity under VSC conditions. This is an important distinction between the ESC and VSC coupling regimes.

**Figure 3:**
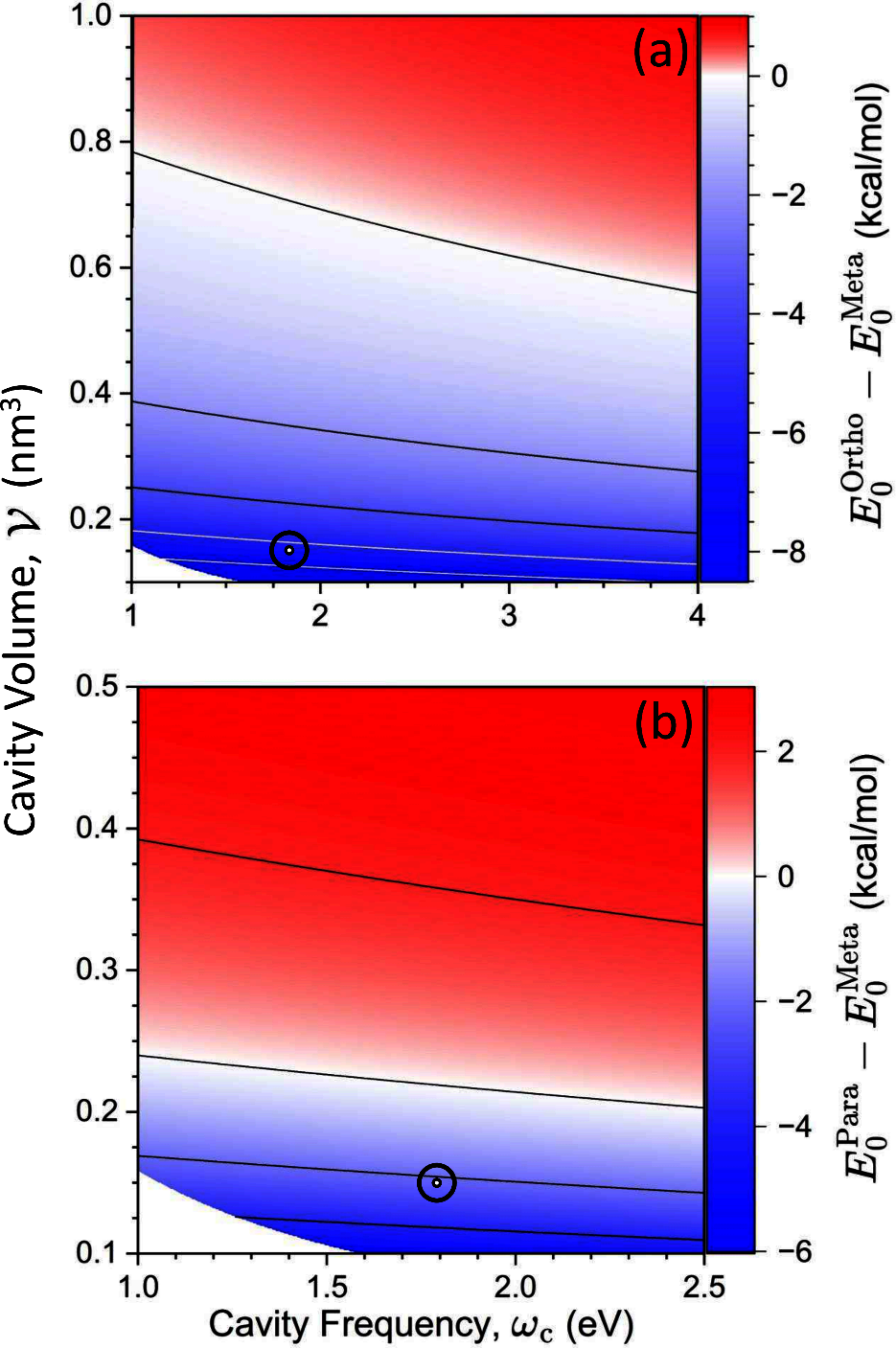
(a) Relative energy of the polaritonic ground states between the *ortho* intermediate and the *meta* intermediate  when the cavity polarization is . (b) Relative energy of the *para* intermediate and the *meta* intermediate  for . These relative energies are reported as functions of the cavity mode volume  and cavity frequency ω_c_. The color scales indicate the relative energy, with red showing thermodynamic favorability for the *meta*-substituted cation and blue for the other. The black open circles correspond to a cavity volume of  = 0.15 nm^3^ and a cavity frequency of ω_c_ = 1.8 eV. This volume corresponds to *A*_0_ = 0.3 a.u. and  a.u.

Figure 3a presents the relative energetics of the *ortho*- and *meta*-substituted intermediates  when the cavity polarization is  (see Figure 1a) as a function of the cavity frequency ω_c_ and the cavity mode volume  (in units of nm^3^). Figure 3b presents the relative energetics of the *para*- and *meta*-substituted intermediates  when  (see Figure 1b). Here, we focus on the range of frequency ω_c_ ≈ 1–4 eV, which is within the typical range possible of nanocavity designs. The cavity frequency of the NPoM cavity depends on the materials of the nanoparticle, the size of the nanoparticle, and the gap size between the particle and the mirror surface. The typical value for a gold nanoparticle is about ω_c_ ≈ 2 eV (600 nm). The typical value for a silver nanoparticle is about ω_c_ ≈ 2.5 eV (500 nm), and that for an aluminum nanoparticle is about ω_c_ ≈ 3 eV (400 nm).^24^

In particular, for the recent experiments^50,51^ on a single emitter strongly coupled to the plasmonic nanocavity, with a gold nanoparticle the cavity has a frequency of ω_c_ = 1.8 eV. Assuming a mode volume equivalent to those previously reported ( nm^3^), the equivalent coupling strength is *A*_0_ = 0.3 a.u. (or λ = 0.1 a.u.), and the field intensity is  V/nm. With these parameters, one can lower the energy of the *ortho* complex by 6.74 kcal/mol compared to the *meta*-substituted intermediate and lower the energy of the *para*-substituted intermediate by 2.24 kcal/mol compared to the *meta*-substituted intermediate. Although coupling to the cavity does not dramatically lower the energy of the *ortho*/*para*-substituted intermediates, these computed coupling strengths indicate favorability for the *ortho* or *para* products, and thus, one should expect to obtain the mixtures of these products together with the *meta*-substituted product.

**Figure 4:**
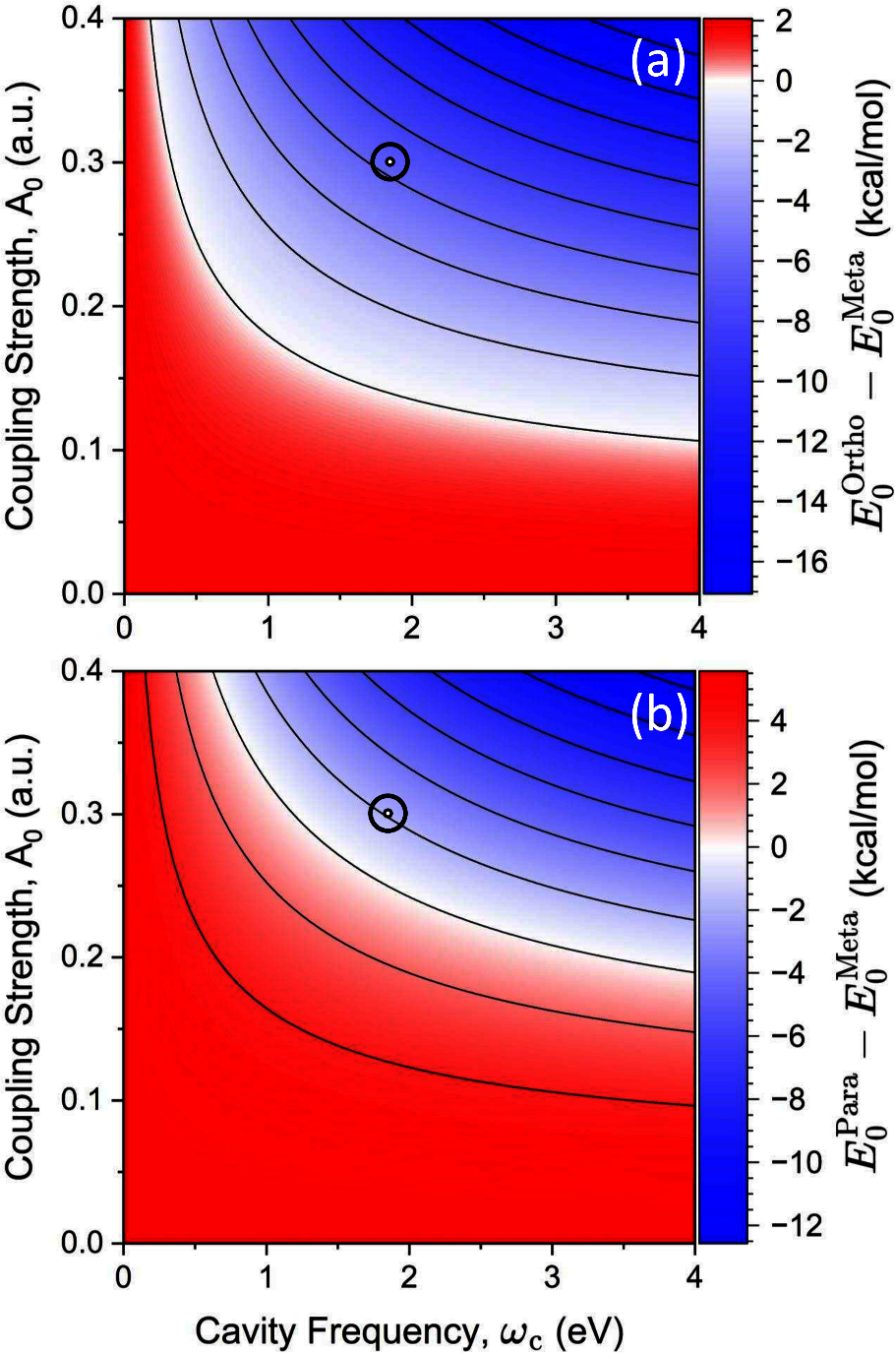
(a) Relative energy of the polaritonic ground states between the *ortho* intermediate and the *meta* intermediate  when the cavity polarization is . (b) Relative energy of the *para* intermediate and the *meta* intermediate  for . These relative energies are reported as functions of the light–matter coupling strength *A*_0_ and cavity frequency ω_c_. The color scales indicate the relative energy, with red showing thermodynamic favorability for the *meta*-substituted cation and blue for the other. The black open circles correspond to a cavity volume of  = 0.15 nm^3^ and a cavity frequency of ω_c_ = 1.8 eV. This volume corresponds to *A*_0_ = 0.3 a.u. and  a.u.

Figure 4 presents the relative stability of the three positively charged intermediate species when the cavity is polarized along the  direction (Figure 4a) or along the  direction (Figure 4b). Similarly to Figure 3, the relative stability is reported as the difference in the polaritonic ground state energies between the *ortho* and *meta* cationic intermediate species in Figure 4a, denoted as , and those between the *para* and *meta* cationic intermediate species in Figure 4b, denoted as . In addition Figure 4 depicts the stability as functions of the cavity frequency ω_c_ and light-matter coupling strength *A_0_*.

The cavity induces a stabilization of the *ortho* (*para*) species in comparison to the *meta* by up to ∼8 kcal/mol (6 kcal/mol) with a cavity frequency of ω_c_ = 4.0 eV (2.5 eV) and a cavity volume of  = 0.1 nm^3^ (0.1 nm^3^). For reference, the volume of a single molecule (roughly the size of benzene) is ∼0.1 nm^3^. The white region indicates the turning point of the cavity mediated selectivity for each polarization. For *ortho*/*meta* selectivity, the cavity is required to have a mode volume of  0.70 nm^3^ (for ω_c_ ≈ 1.8 eV) in order to favor *ortho* selectivity. For *para* to be the favored product over *meta*, the cavity is required to have (for ω_c_ ≈ 1.8 eV) a mode volume of  0.225 nm^3^. In both panels, the open circles indicate currently accessible cavity parameters based on state-of-the-art NPoM plasmonic cavities^24^ and correspond to a cavity volume of  nm^3^ and a cavity frequency of ω_c_ = 1.8 eV. At these parameters and in both polarizations, we predict that the cavity is already able to provide the selectivity of this bromination reaction away from the expected *meta* product. Thus, our theoretical predictions should be experimentally realizable with current experimental cavity designs. The typical single molecule cavity setup in experiments is constructed by assembling an ensemble of gold nanoparticles on top of a gold mirror, creating a small confinement and large field strength between each nanoparticle and Au surface.^50^ Thus, there will be a large ensemble of Au particle–molecule hybrid systems on the gold surface.^50^ Experimentally, one should be able to characterize the non-*meta* product.

As an important note for the experimental design of cavities, we want to emphasize that light–matter resonance effects are not the primary mechanisms in these cavity induced modifications (Figures 3 and 4). The cavity frequency only plays a role in optimizing the magnitude of the light–matter coupling strength (eq 1). From an experimental perspective, this implies that one does not need to design the cavity with a frequency to match certain optical transitions. We expect this feature of ground state modifications via cavity quantum electrodynamics to alleviate the usual experimental difficulties associated with tuning the resonance condition between the cavity and the molecular absorption frequencies.

## Conclusions

In this work, we used the *ab initio* cavity quantum electrodynamics (QED) approach to investigate a chemical reaction, the bromination of nitrobenzene, coupled to an optical cavity. Our approach is based on the previously developed parametrized QED (pQED) method, which uses the QED Pauli–Fierz Hamiltonian to describe light and matter interactions and uses adiabatic electronic states and all dipole matrix elements between them as inputs to compute the polariton eigenenergies.^16^

The bromination of nitrobenzene exhibits near 100% selectivity, favoring the *meta*-substituted isomer. Upon coupling to the cavity, we theoretically calculated the relative energies of the *meta*-, *ortho*-, and *para*-substituted cationic intermediates BrC_6_H_4_NO2^+^, which are key intermediates that dictate the outcome of the reaction. Outside the cavity, the *meta*-substituted intermediate is 2 kcal/mol lower than the *ortho*-substituted intermediate and about 5 kcal/mol lower than the *para*-substituted intermediate, in agreement with reported experimental results. Upon coupling to the cavity and aligning the cavity polarization direction along  (see Figure 1a), the *ortho*-substituted intermediate is energetically more stable than the *meta*-substituted intermediate by up to 6 kcal/mol for a cavity frequency ω_c_ and cavity volume  chosen to match state-of-the-art plasmonic cavity designs.^24^ When the cavity polarization direction is along  (see Figure 1b), the *para*-substituted intermediate is energetically more stable than the *meta*-substituted intermediate, with up to −2 kcal/mol. These changes in the selectivity of the various substituted intermediates are due to the quantum light–matter interactions between the molecules and cavity, which mixes the character of electronic excited states into the polariton ground state. These changes were characterized by using the electronic density difference of the system inside and outside the cavity. We thus have theoretically shown that one can obtain *ortho*- or *para*-substituted bromonitrobenzene when coupling the reaction to an optical cavity, flipping the selectivity compared to outside the cavity.

To probe the possibility of experimental realization of our theoretical prediction, we focused on the currently available plasmonic nanocavity parameters for the cavity frequency and field strength. We further scanned all possible polarization directions. Interestingly, we can find finite regions in the configuration space of polar angle and azimuthal angle that make the *ortho*- or *para*-substituted species more stable than the *meta*-substituted intermediate, with the largest stabilization energy of 6.43 kcal/mol (11.24 *k*_B_*T* at room temperature) for *ortho* and 1.97 kcal/mol (3.42 *k*_B_*T*) for *para*. The relative probability of forming the *ortho*- and *para*-substituted species is  = ∼7 × 10^5^ for ortho and  for *para*. This implies that with the nanocavity and fully isotropic orientations of the molecule inside, one should expect to generate a non-*meta*-substituted product (in addition to the usual *meta* species), demonstrating that coupling to the cavity can make the nonstandard product, which cannot be easily obtained otherwise. More importantly, we have explicitly shown that coupling to a photonic cavity will dramatically change the selectivity. Furthermore, from an experimental perspective, detection of these anomalous products will provide conclusive evidence of the cavity mediated effects in the ground state.

From a synthetic perspective, coupling to the cavity flips the selectivity expected for electrophilic aromatic substitution in two important and useful ways. First, it flips the normal selectivity of the nitrogroup to make it an *ortho*/*para* director. Second, this approach appears to stabilize *ortho* substitution over *para* substitution. As noted above, this is also a rare selectivity that would complement existing approaches well.^56^

Additionally, we note that there are no obvious resonance effects in the observables presented in this work. This is because the molecule interacts through nonresonant terms such as the DSE and the indirect bilinear interactions (with examples discussed in the Theoretical Methods section). Further, when coupling *N* > 1 molecules with the cavity, there will be upper and lower polaritonic states, together with a dense manifold of “dark” states that contain negligible amounts of photonic character.^1,57^ While these dark states have been suggested to be important for excited-state dynamics, the exploration in the current work is focused on ground-state reactivities on the ground polariton state. Thus, the formation of dark states will have minimal impact on the cavity modified reactivities presented here.

Overall, this work demonstrates the possibility of polariton-mediated changes to the selectivity of well-known chemical reactions. The theoretical prediction can, in principle, be experimentally verified using state-of-the-art plasmonic nanocavity designs.

## Theoretical Methods

We use the *ab initio* polariton approach we developed in a previous work, which we refer to as the pQED approach.^16^ The pQED approach uses the Pauli–Fierz Hamiltonian in the Born–Oppenheimer approximation (see eq 2) to describe light and matter interactions and uses adiabatic electronic states as the basis for the electronic degrees of freedom and Fock states as the basis for the photonic degree of freedom.

The light–matter interaction Hamiltonian under the dipole gauge^1,9,29^ is expressed as
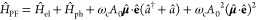
2where  is the electronic Hamiltonian under the Born–Oppenheimer approximation (i.e., without the nuclear kinetic energy operator),  is the Hamiltonian of the cavity field,  and  are the raising and lowering operators of the cavity field, respectively,  is a unit vector indicating the field polarization direction, and  is the dipole operator of the molecule, including, for example, permanent dipole , transition dipole  and all other possible dipole matrix elements. Through the light–matter coupling terms in eq 2, various photon-dressed electronic states will be coupled to each other. For example, |ψ_g_, 1⟩ ≡ |ψ_g_⟩ ⊗ |1⟩ (the ground electronic state with 1 photon) and |ψ_e_, 0⟩ ≡ |ψ_e_⟩ ⊗ |0⟩ (an excited electronic state with 0 photons) will couple through , where *μ*_ge_ is the transition dipole between the ground state and excited state projected along the  direction. When the energy of these two basis states become close, the |ψ_g_, 1⟩ and |ψ_e_, 0⟩ states hybridize, leading to the formation of excited polariton states. This is the typical resonant light–matter coupling induced hybridization and generating new eigenstates and polaritons.

The direct modification of polariton ground states can be caused by two other physical processes:^1,9,31^ (i) off-resonance light–matter interactions (third term in eq 2) through the ground-state permanent dipole and optical transition dipoles between the ground and excited states and (ii) a dipole self-energy (DSE) term (final term in eq 2). For example, in (i), similarly to above, |ψ_g_, 0⟩ will couple to the |ψ_g_, 1⟩ state through a term proportional to , and |ψ_g_, 1⟩ will couple to |ψ_e_, 0⟩ through . Importantly, note that there may be many such electronic excited states ψ_e_ that contribute to the ground state through these off-resonant interactions (see the Supporting Information for details on the specific interaction terms depicted through an analysis of the ground-state density matrix). In (ii), the DSE term allows for extensive coupling through the square of the electronic dipole matrix , where we denote  as the projection of  along the cavity polarization direction . The matrix elements between the ground state |ψ_g_⟩ and any electronic state |ψ_α_⟩ due to the DSE coupling can be expressed as , where α and γ include the ground and excited electronic states. The direct coupling (i) is responsible for the accumulation of photons in the ground state,^10^ while DSE (ii) is largely responsible for the modifications to the ground-state energy.^58^

The polariton eigenstates and eigenenergies are obtained by solving the following equation:
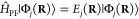
3where  is given in eq 2, *E*_*j*_(**R**) is the Born–Oppenheimer polaritonic potential energy surfaces (which parametrically depend on the nuclear coordinates **R**), and |*E*_*j*_(**R**)⟩ is the polariton state. We directly diagonalize the polaritonic Hamiltonian  matrix and obtain the eigenvalues. The basis is constructed using the tensor product of electronic adiabatic states |ψ_α_(**R**)⟩ (i.e., the eigenstates of the electronic Hamiltonian ) and the Fock states |*n*⟩ (i.e., the eigenstates of the photonic Hamiltonian ), expressed as |ψ_α_(**R**)⟩ ⊗ |*n*⟩ ≡ |ψ_α_(**R**), *n*⟩. This basis is used to evaluate the matrix elements of , and diagonalizing it provides *E*_*j*_(**R**) and the corresponding polariton states
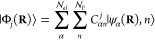
4where . Here, the number of included electronic states, , and photonic Fock/number states, , are treated as convergence parameters. A convergence test is provided in the Supporting Information, and the typical numbers of states are  and . Further details regarding the pQED approach are provided in the Supporting Information. The accuracy of the above-described pQED approach has been benchmarked^16^ with the more accurate self-consistent QED coupled-cluster (scQED-CC) approach, where the pQED method generates nearly quantitative agreement with the scQED-CC approach.

The observations presented here, including the DSE mentioned above, go beyond the often-used classical field Hamiltonian^59−62^
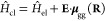
5where **E** is the classical electric field, which has also been shown to modify ground-state reactivities.^59−62^ Note that  only includes a frequency independent field  that couples only to the ground-state permanent dipole ***μ***_gg_ of the molecule. Often, the energy of the classical Hamiltonian can be fit to a linear model: .^59^ In this case, the changes to transition state barrier heights, for example, depend on the difference in the permanent dipole moments between the reactant and transition-state geometries:^59,62^ ΔΔ*E*_class_ ∝ Δ*μ*_gg_ = *μ*_gg_(**R**_TS_) – *μ*_gg_(**R**_React_). As discussed above, the QED Pauli–Fierz Hamiltonian includes many other effects, such as the direct coupling between light and matter through the third term in eq 2 (resonant and off-resonant contributions), which accounts for all possible dipole matrix elements and the DSE term (fourth term in eq 2) that strongly couples the electronic states through the square of the total dipole operator  and is thus mixing all of the electronic states. Additionally, others have noted that the inclusion of the DSE term in the Hamiltonian results in a guaranteed ground state due to the quadratic confinement of the DSE term in the dipole gauge.^1,29,58,63,64^ It is thus interesting to find the distinct difference between the classical field effect and the QED effect on ground-state modification in the future.

Finally, we want to mention that the DSE has the dominant contribution to the ground-state energy modifications in this work. In Figure S1 in the Supporting Information, we have analyzed energy contributions from all terms in the Pauli–Fierz QED Hamiltonian, and DSE closely follows the changes of the total energy given by the full Pauli–Fierz Hamiltonian, and all other terms cancel. Alternatively, the DSE effect can be understood from the self-consistent treatment of the *ab initio* QED^11^ from considering the mean-field approach (i.e., QED Hartree–Fock) to the Pauli–Fierz Hamiltonian. From this perspective and in the coherent state representation of the cavity mode,^11,65^ the bilinear light–matter coupling term can be shifted away,^1^ leaving only the DSE term, representing the dipole fluctuations , where . From this mean-field electronic perspective, cavity induced modifications only arise due to the DSE term itself. However, the pQED approach we used in the current work goes well beyond the mean-field level due to the exact diagonalization of the Pauli–Fierz Hamiltonian.^16^

### Computational Details

All electronic structure calculations were performed using the QCHEM software package^66^ using linear response time-dependent density functional theory (LR-TD-DFT) and the ωB97XD hybrid exchange-correlation functional with the 6-311+G* basis set. The geometries of the BrC_6_H_4_NO_2_^+^ intermediate with various substitution positions are optimized in its electronic ground states. For cavity polarization  along a particular direction with (θ, ϕ) angles, the  term in eq 2 is evaluated as follows:
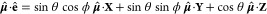
6where , , and  are the dipole operators projected along the **X**-, **Y**-, and **Z**-directions, respectively. The matrix elements of these projected dipole operators, , , and , are obtained from electronic structure calculations and are used to evaluate the  term in eq 2. The electronic excited-state energies and the molecular transition dipole matrix were computed using the QCHEM package.^66^

## Supporting Information Available

Details of theoretical approaches, theoretical analysis of the energy contributions for polariton ground state, additional results on density difference, convergence of the calculation with adiabatic-Fock basis, additional analysis of the ground-state density matrix, and additional results on cavity polarization (PDF)
