# Supplementary material for: Cavity Quantum Electrodynamics Enables para- and ortho-Selective Electrophilic Bromination of Nitrobenzene
Source: J Am Chem Soc. 2024 May 30;146(23):16184–93. doi: 10.1021/jacs.4c04045 (PMC11177318; doi:10.1021/jacs.4c04045)
Supplement: Supplementary file 1 — ja4c04045_si_001.pdf [file ja4c04045_si_001.pdf]

Cavity Quantum Electrodynamics Enables *para*- and *ortho*- Selective Electrophilic Bromination of Nitrobenzene

Braden M. Weight,[*,†] Daniel J. Weix,[*,‡] Zachary J. Tonzetich,[¶] Todd D. Krauss,[§,‖] and Pengfei Huo[*,§,‖]

†*Department of Physics and Astronomy, University of Rochester, Rochester, NY 14627, U.S.A.*

‡*University of Wisconsin-Madison, Madison, WI 53706, U.S.A.*

¶*Department of Chemistry, University of Texas at San Antonio, San Antonia, TX 78249, U.S.A.*

§*Department of Chemistry, University of Rochester, Rochester, NY 14627, U.S.A.*

‖*The Institute of Optics, Hajim School of Engineering, University of Rochester, Rochester, NY 14627, U.S.A.*

E-mail: bweight@ur.rochester.edu; dweix@wisc.edu; pengfei.huo@rochester.edu

Theoretical Approaches

Electronic Hamiltonian. Here, we follow our previous work[1] to compute ab initio polariton states. The molecular Hamiltonian $\hat{H}_{\mathrm{M}}$ can be written in terms of the nuclear kinetic energy operator $\hat{T}_R$ and the electronic Hamiltonian $\hat{H}_{\mathrm{el}}$ as,

$$\hat{H}_{\mathrm{M}} = \hat{T}_R + \hat{H}_{\mathrm{el}} = \hat{T}_R + \hat{T}_r + \hat{V}, \tag{S1}$$

where $\hat{T}_r$ is the electronic kinetic energy operator and $\hat{V}(\mathbf{R}) = \hat{V}_{\mathrm{NN}} + \hat{V}_{\mathrm{eN}} + \hat{V}_{\mathrm{ee}}$ is the electronic potential including the nuclear-nuclear $\hat{V}_{\mathrm{NN}}$, electron-nuclear $\hat{V}_{\mathrm{eN}}$, and electron-electron $\hat{V}_{\mathrm{ee}}$ interactions. The electronic Hamiltonian $\hat{H}_{\mathrm{el}} = \hat{H}_{\mathrm{M}} - \hat{T}_R$ is routinely diagonalized via standard electronic structure packages, which attempt to solve the following eigenvalue

problem,

$$\hat{H}_{\text{el}}|\psi_\alpha(\mathbf{R})\rangle = E_\alpha(\mathbf{R})|\psi_\alpha(\mathbf{R})\rangle, \tag{S2}$$

which defines the adiabatic electronic states $|\psi_\alpha(\mathbf{R})\rangle$ and potential energy surfaces $E_\alpha(\mathbf{R})$ for the α_{th} state. Note that both the eigenvalues and eigenfunctions are parameterized by the nuclear positions in the Born-Oppenheimer approximation.

The coupling between light and matter, as we will see later, is mediated via the molecular dipole operator $\hat{\boldsymbol{\mu}} = \sum_i z_i \hat{\mathbf{R}}_i - \sum_k \hat{\mathbf{r}}_k$ and the quantized electric field of the optical cavity $\hat{\mathbf{E}}$. The matrix elements of the dipole operator in the adiabatic basis can be written as,

$$\mu_{\alpha\beta}(\mathbf{R}) = \langle\psi_\alpha(\mathbf{R})|\hat{\mu}|\psi_\beta(\mathbf{R})\rangle. \tag{S3}$$

For large systems with many electrons, the maximum number of electronic states becomes impractically large for standard electronic structure calculations, even considering only the single excitation manifold. Further, for single-reference methods, such as linear response time-dependent density functionally theory (LR-TD-DFT), the efficient *and accurate* calculation of high-energy excited states is not always trustworthy, so in practice we employ a smaller Hilbert space for the subsequent calculations of polaritonic states than that implied by the total electronic Hamiltonian $\hat{H}_{\text{el}}$ in Eq. S2. This smaller space can be defined through the following projection operator,

$$\hat{\mathcal{P}} = \sum_{\alpha=0}^{\mathcal{N}_{\text{el}}-1} |\psi_\alpha(\mathbf{R})\rangle\langle\psi_\alpha(\mathbf{R})|, \tag{S4}$$

where $\mathcal{N}_{\text{el}}$ is the number of included adiabatic electronic states (ordered by increasing energy). The identity operator for the total Hilbert space can be written as $\hat{\mathcal{I}}_{\text{el}} = \hat{\mathcal{P}} + \hat{\mathcal{Q}}$, where $\hat{\mathcal{Q}}$ is composed of all non-included states. The projected electronic Hamiltonian can be written as,

$$\hat{\mathcal{P}}\hat{H}_{\text{el}}\hat{\mathcal{P}} = \sum_{\alpha=0}^{\mathcal{N}_{\text{el}}-1} E_\alpha(\mathbf{R})|\psi_\alpha(\mathbf{R})\rangle\langle\psi_\alpha(\mathbf{R})| \tag{S5}$$

For the remainder of this work, it will be assumed that all Hamiltonians and operators reside in the truncated Hilbert space $\hat{\mathcal{P}}\hat{H}_{\mathrm{M}}\hat{\mathcal{P}} \to \hat{H}_{\mathrm{M}}$.

Pauli-Fierz QED Hamiltonian. We use the Pauli-Fierz (PF) Hamiltonian $\hat{H}_{\mathrm{PF}}$ to model the interactions between the molecular and photonic degrees of freedom inside an optical or plasmonic cavity. The PF Hamiltonian is expressed as

$$\begin{aligned}\hat{H}_{\mathrm{PF}} &= \hat{H}_{\mathrm{M}} + \hat{H}_{\mathrm{ph}} + \hat{H}_{\mathrm{el-ph}} + \hat{H}_{\mathrm{DSE}} \\ &= \hat{H}_{\mathrm{M}} + \hbar\omega_{\mathrm{c}}(\hat{a}^{\dagger}\hat{a} + \frac{1}{2}) + \omega_{\mathrm{c}}\mathbf{A_0}\cdot\hat{\boldsymbol{\mu}}(\hat{a}^{\dagger} + \hat{a}) + \frac{\omega_{\mathrm{c}}}{\hbar}(\mathbf{A_0}\cdot\hat{\boldsymbol{\mu}})^2\end{aligned} \tag{S6}$$

where ω_{c} is the cavity frequency and $\hat{a}^{\dagger}$ ($\hat{a}$) is creation (annihilation) ladder operator for the photon field. The Hamiltonian can be factored into the form of a shifted harmonic oscillator via the definition of the canonical coordinates (*i.e.* the operators of positions and momentum) of the quantum harmonic oscillator: $\hat{q}_{\mathrm{c}} = \sqrt{\frac{\hbar}{2\omega_{\mathrm{c}}}}(\hat{a}^{\dagger} + \hat{a})$ and $\hat{p}_{\mathrm{c}} = \sqrt{\frac{\hbar\omega_{\mathrm{c}}}{2}}(\hat{a}^{\dagger} - \hat{a})$. Further, $\mathbf{A_0} = \sqrt{\frac{1}{2\omega_{\mathrm{c}}\varepsilon\mathcal{V}}}\ \hat{\mathbf{e}} = A_0 \cdot \hat{\mathbf{e}}$ is the light-matter coupling strength, taken as the vector potential with polarization along $\hat{\mathbf{e}}$. Another common choice is $\boldsymbol{\lambda}$ and is related to $\mathbf{A_0}$ as $\mathbf{A}_0 = \sqrt{\frac{1}{2\omega_{\mathrm{c}}\varepsilon\mathcal{V}}}\ \hat{\mathbf{e}} = \sqrt{\frac{1}{2\omega_{\mathrm{c}}}}\ \boldsymbol{\lambda}$, where $\mathcal{V}$ is the quantization volume of the photon field, ε is the electric permittivity, and $\hat{\mathbf{e}}$ is the unit vector of the electric field. Eq. S6 is composed of four main elements: the molecular Hamiltonian $\hat{H}_{\mathrm{M}}$, the photonic Hamiltonian $\hat{H}_{\mathrm{ph}}$, the light-matter interaction $\hat{H}_{\mathrm{el-ph}}$, and the dipole self-energy $\hat{H}_{\mathrm{DSE}}$.

Similarly to the electronic Hamiltonian (Eq. S2), we wish to solve an eigenvalue problem without the nuclear kinetic energy operator $\hat{T}_{R}$, which we define the polaritonic Hamiltonian $\hat{H}_{\mathrm{pl}}$ (analogously to the electronic Hamiltonian $\hat{H}_{\mathrm{el}}$) as,

$$\hat{H}_{\mathrm{pl}} = \hat{H}_{\mathrm{PF}} - \hat{T}_{\mathrm{R}} = \hat{H}_{\mathrm{el}} + \hat{H}_{\mathrm{ph}} + \hat{H}_{\mathrm{el-ph}} + \hat{H}_{\mathrm{DSE}}, \tag{S7}$$

whose eigenvalue equation can be written as,

$$\hat{H}_{\mathrm{pl}}|\Phi_j(\mathbf{R})\rangle = E_j(\mathbf{R})|\Phi_j(\mathbf{R})\rangle, \tag{S8}$$

where $E_j(\mathbf{R})$ are the Born-Oppenheimer polaritonic potential energy surfaces and $|E_j(\mathbf{R})\rangle$ are the adiabatic polaritonic states.

Adiabatic-Fock Basis. One can directly diagonalize QED Hamiltonian, Eq. S7, in the basis of electronic adiabatic states $|\psi_\alpha(\mathbf{R})\rangle$ (defined in Eq. S2) paired with the vacuum Fock states for the photonic DOFs $|n\rangle$ (eigenvectors of $\hat{H}_{\mathrm{ph}}$ in Eq. S6). The polaritonic states are then represented as linear combinations of the adiabatic-Fock basis states (with contracted notation $|\psi_\alpha(\mathbf{R})\rangle \otimes |n\rangle = |\psi_\alpha(\mathbf{R}), n\rangle$) as,

$$|\Phi_j(\mathbf{R})\rangle = \sum_{\alpha}^{\mathcal{N}_{\mathrm{el}}} \sum_{n}^{\mathcal{N}_{\mathrm{F}}} C^j_{\alpha n} |\psi_\alpha(\mathbf{R}), n\rangle, \tag{S9}$$

where $C^j_{\alpha n} = \langle \psi_\alpha(\mathbf{R}), n | \Phi_j(\mathbf{R})\rangle$. Here, $\mathcal{N}_{\mathrm{el}}$ and $\mathcal{N}_{\mathrm{F}}$ are treated as convergence parameters such that the polaritonic observables are adequately converged.

Density Difference. The primary analysis technique used in this work is the difference density function for the ground state defined as,

$$\Delta\rho_{00}(\mathbf{r}) = \rho^{\mathrm{M}}_{00}(\mathbf{r}) - \xi_{00}(\mathbf{r}) \tag{S10}$$

where $\rho^{\mathrm{M}}_{00}(\mathbf{r})$ is the one-particle ground state polaritonic density where the photonic DOFs have been traced out, and

$$\xi_{\alpha\beta}(\mathbf{r}) = \psi^*_\alpha(\mathbf{r})\psi_\beta(\mathbf{r}) \tag{S11}$$

is the one-particle electronic density ($\alpha = \beta$) or transition density ($\alpha \neq \beta$).[1] The polaritonic ground state density can be expanded in the adiabatic-Fock basis as,

$$\hat{\rho}^{\mathrm{M}}_{00} = \mathrm{Tr}_{\mathrm{ph}}\big[\hat{\rho}_{00}\big] = \mathrm{Tr}_{\mathrm{ph}}\big[|\Phi_0\rangle\langle\Phi_0|\big] = \sum_{\alpha\beta}^{\mathcal{N}_{\mathrm{el}}} \sum_{n}^{\mathcal{N}_{\mathrm{F}}} \; C^0_{\alpha n}\; C^0_{\beta n}\; |\psi_\alpha\rangle\langle\psi_\beta|, \tag{S12}$$

where we have made use of the orthogonality of the vacuum Fock states, $\langle n|m\rangle = \delta_{nm}$. Note that $\hat{H}_{\mathrm{pl}}$ is purely real, so $(C^0_{\alpha n})^* = C^0_{\alpha n}$.

In this case, the one-particle ground state polaritonic density is composed of linear combinations of *all possible* one-particle electronic density matrices, including state-densities $\xi_{\alpha\alpha}(\mathbf{r})$, ground-to-excited transition densities $\xi_{0\alpha}(\mathbf{r})$ and $\xi_{\alpha 0}(\mathbf{r})$, and excited-to-excited transition densities $\xi_{\alpha\beta}(\mathbf{r})$. The volumetric grid data for the bare electronic state densities $\xi_{\alpha\alpha}(\mathbf{r})$ and ground-to-excited transition densities $\xi_{\alpha 0}(\mathbf{r})$ were computed using the QCHEM package.[2] In principle, the excited-to-excited transition densities $\xi_{\alpha\beta}(\mathbf{r})$ can be computed using the Z-vector method[3–5] but were not included in this work. This is allowed since the contributions $C^0_{\alpha\beta}$ of these electronic densities to the ground state polaritonic density are small (smallest terms $C^0_{\alpha\beta} \sim \sum_n C^0_{\alpha n}\, C^0_{\beta n}$, with $\alpha \neq \beta$) compared to the larger ground-to-excited transition density (largest terms $C^0_{\alpha 0} = C^0_{0\alpha} \sim \sum_n C^0_{0n}\, C^0_{\alpha n}$) and the state densities (second largest terms $C^0_{\alpha\alpha} \sim \sum_n |C^0_{\alpha n}|^2$) since $C^0_{\alpha 0}$ is always large.

The integrated difference density was computed (Figs. 2 and S3) as,

$$\Delta\rho_{00}(x, y) = \int dz\ \Delta\rho_{00}(x, y, z). \tag{S13}$$

Computational Details. All electronic structure calculations were performed using the QCHEM software package[2] with linear response time-dependent density functional theory (LR-TD-DFT), and the ωB97XD hybrid exchange-correlation functional with the 6-311+G* basis set. The electronic excited state energies, the molecular transition dipole matrix (Eq. S3), and the volumetric grid data for the bare electronic state densities $\xi_{\alpha\alpha}(\mathbf{r})$ and ground-to-excited transition densities $\xi_{0\alpha}(\mathbf{r})$ were all computed using the QCHEM package.[2]

Theoretical Analysis of the Energy Contributions for Polariton Ground State

It is theoretically insightful to examine the contributions from each term in the Hamiltonian (Eq. 2 of the main text) to understand the change of the preference for the reactive intermediate. In Fig. S1, we plot the energy difference $E_a^{ortho}(\mathbf{R}) - E_a^{meta}(\mathbf{R})$ (Fig. S1a) and $E_a^{para}(\mathbf{R}) - E_a^{meta}(\mathbf{R})$ (Fig. S1b) as functions of the light-matter coupling strength A_0. The individual energy contributions are calculated as $E_a = \langle \Phi_0 | \hat{H}_a | \Phi_0 \rangle$ where $\hat{H}_a \in (\hat{H}_{\mathrm{PF}}, \hat{H}_{\mathrm{el}}, \hat{H}_{\mathrm{ph}}, \hat{H}_{\mathrm{el-ph}}, \hat{H}_{\mathrm{DSE}})$ and $|\Phi_0\rangle$ is the ground polaritonic state (see Eq. S8). The cavity frequency is $\omega_{\mathrm{c}} = 1.8$ eV with cavity polarization (Fig. S1a) $\hat{\mathbf{e}} = (\theta_1, \phi_1)$ and (Fig. S1b) $\hat{\mathbf{e}} = (\theta_2, \phi_2)$. For each case, the total ground state energy difference (thick grey curve) is shown alongside E_{el} (black), E_{ph} (orange), $E_{\mathrm{el-ph}}$ (blue), and E_{DSE} (red). Furthermore, the sum of the light-matter interaction and dipole self-energy contributions, $E_{\mathrm{el-ph}} + E_{\mathrm{DSE}}$, are shown (dashed purple).

For both cases, the trends in individual energies are similar. The bare electronic (black) and photonic (orange) energies contribute negatively to the energy difference by $\sim 4-6$ and ~ 1 kcal/mol, respectively. Further, the light-matter coupling term (blue) always contributes positively to the energy difference by $\sim 6-8$ kcal/mol. Finally, the DSE (red) contributes negatively to the energy difference while also having the largest magnitude contribution at $\sim 12-15$ kcal/mol. The most interesting feature is the cancellation of the individual energy contributions. For example, the total energy (thick grey curve) closely follows the contribution from the DSE, implying that the other three terms in the Hamiltonian largely cancel among themselves. Overall, these individual energy contributions confirm that the major contributions to the ground state changes stem from the DSE, in agreement with the early works.[6,7]

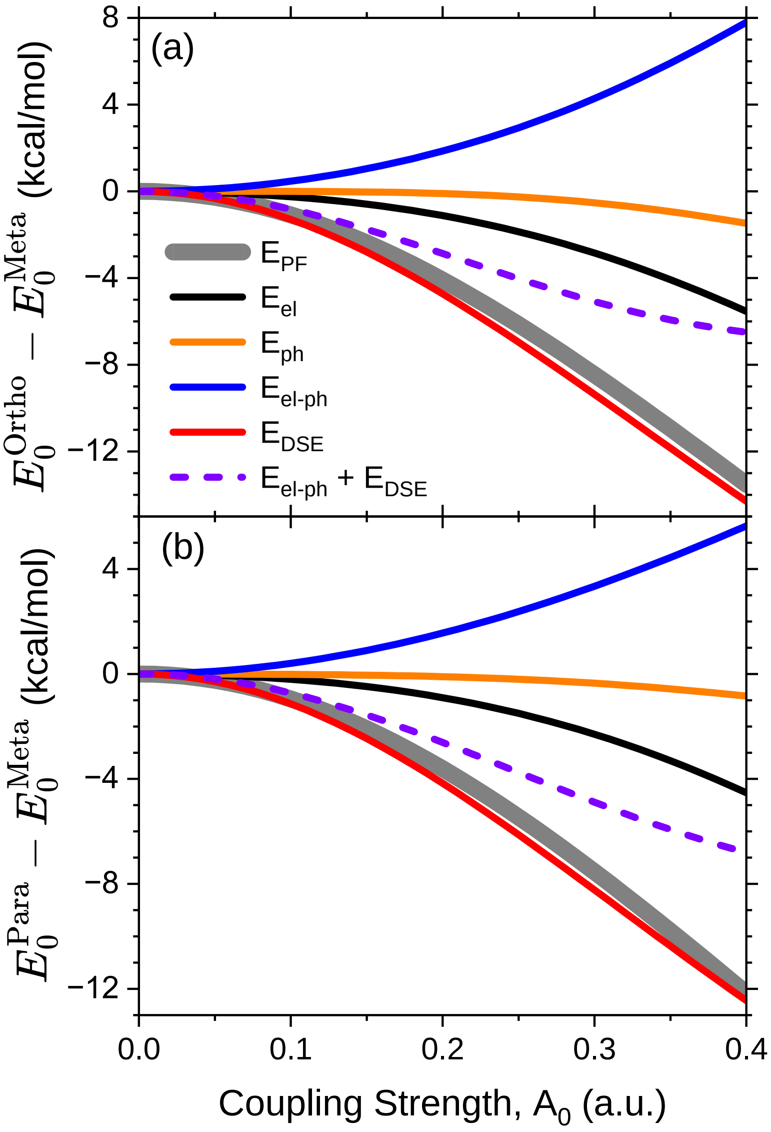

Figure S1: Contributions of the four Hamiltonian terms to the ground state total energy difference between intermediates. The partial energy is defined as $E_a = \langle E_0|\hat{H}_a|E_0\rangle$ where $\hat{H}_a \in (\hat{H}_{\mathrm{PF}}, \hat{H}_{\mathrm{el}}, \hat{H}_{\mathrm{ph}}, \hat{H}_{\mathrm{el-ph}}, \hat{H}_{\mathrm{DSE}})$ and $|E_0\rangle$ is the ground polaritonic stated defined as $\hat{H}_{\mathrm{PF}}|E_0\rangle = E_0|E_0\rangle$ via Eq. S8. The energy difference between the (a) *ortho/meta* intermediates is $E_a^{ortho}(\mathbf{R}) - E_a^{meta}(\mathbf{R})$ and (b) *para/meta* is $E_a^{para}(\mathbf{R}) - E_a^{meta}(\mathbf{R})$. The cavity frequency is $\omega_{\mathrm{c}} = 1.8$ eV with cavity polarization (a) $\hat{\mathbf{e}} = (\theta_1, \phi_1)$ and (b) $\hat{\mathbf{e}} = (\theta_2, \phi_2)$, respectively. Note that in both panels the electronic contribution $\langle E_0|\hat{H}_{\mathrm{el}}|E_0\rangle = E_{\mathrm{el}}$ was vertically shifted such that $E_{\mathrm{el}} = 0$ (as the reference potential) at $A_0 = 0.0$ a.u.

Additional Results on Density Difference

We begin by examining the bare nitrobenzene reactant molecule. While this species is not the primary interest of this work, its high symmetry allows for a simple introduction to the

tools and explanations used in the more complicated brominated intermediate species of the reaction. Fig. S2 presents the difference density contour of the nitrobenzene species with the cavity polarized along the (Fig. S2a) X- and (Fig. S2b) Y-directions (see cartesian axes above each panel). The difference density is defined as $\Delta\rho_{00}(x, y, z) = \rho_{00^{\mathrm{M}}}(x, y, z) - \xi_{00}(x, y, z)$, where $\rho_{00}(x, y, z)$ is the polaritonic ground state density after tracing out the photonic DOFs and $\xi_{00}(x, y, z)$ is the bare electronic ground state density. See Eqs. S10-S13 for theoretical details on the difference density. The two-dimensional contour map was constructed by integrating out the Z-component as $\Delta\rho_{00}(x, y) = \int\ dz\ \Delta\rho(x, y, z)$ (see Eq. S13). The color bar indicates the sign and magnitude of the redistribution of electronic density upon insertion into the cavity. Red color indicates electron accumulation and blue color indicates electron depletion. In both cases of polarization, the light-matter coupling strength $A_0 = 0.3$ a.u. with cavity frequency $\omega_\mathrm{c} = 1.8$ eV. The difference density arises, physically, since the cavity induces a mixing of the electronic states (more specifically, mixes electronic transition densities) into the ground state, thus allowing for a modification of the character and, hence, the electronic distribution/localization. In this sense, the cavity allows for the charge to transfer between atoms and bonds (or more generally between single-particle orbitals) upon insertion into the cavity.

Fig. S2a shows that when the cavity is polarized along the X- direction (primary axis of the molecule), *i.e.*, along the strong permanent dipole moment attributed to the nitro group, the magnitude of the difference density (and therefore the charge reorganization) is larger than when the cavity is polarized along the perpendicular Y-direction (Fig. S2b) by a factor of 2. Furthermore, in both cases of polarization, the relative accumulation and depletion maxima and minima are roughly symmetric, ± 6 and ± 3 m|e|, respectively, where m|e| is 1000× |e| and |e| is the charge of one electron.

It is now prudent to examine the differences in the spatial localization of electron density accumulation or depletion between the two polarizations. The X-polarized cavity (Fig. S2a) induces a strong depletion of electron density at the oxygen atoms as well as at the nitrogen-

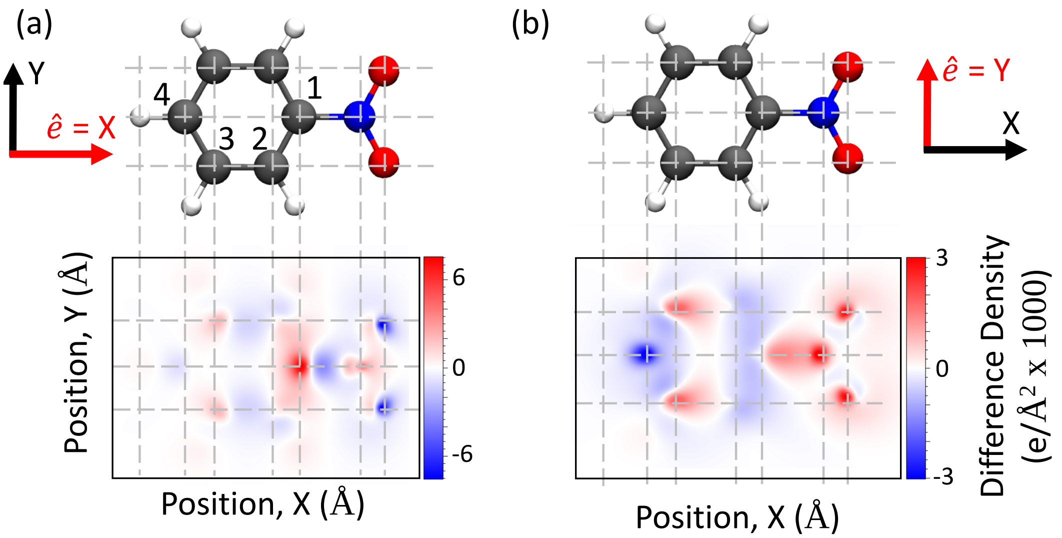

Figure S2: Ground state density difference $\Delta\rho_{00}(x, y) = \int dz[\rho_{00}^{\mathrm{M}}(x, y, z) - \xi_{00}(x, y, z)]$ of the nitrobenzene reactant species with the cavity polarization along the (a) X- and (b) Y-directions. The light-matter coupling strength $A_0 = 0.3$ a.u. and cavity frequency $\omega_{\mathrm{c}} = 1.8$ eV. The color bar indicates the magnitude and sign of the difference density $\Delta\rho_{00}(x, y)$, where positive (red) indicates electron charge accumulation and negative (blue) indicates electron charge depletion upon coupling the molecule with the cavity. Note that the color bar scales in panel (a) and panel (b) are different.

carbon bond. Further, there is weak electron depletion at the 2,3-bonds of the benzene ring, the *ortho*-carbons, and at the *para*-carbon. The accumulation follows a similar trend but at the opposing spatial regions of the molecule: strong accumulation occurs at the nitrogen-connected carbon and weak accumulation at the nitrogen, nitrogen-oxygen bonds, and the *meta*-carbons.

There are also significant differences in how the charge is rearranged by different cavity polarization directions by examining Fig. S2. (i) The 1,2-bonds are largely depleted in the Y-polarized cavity while showing a weaker accumulation at the nitrogen-connected carbon atom. (ii) The oxygen atoms are accumulating electronic density in Y-polarization, and depleting electron density in X-polarization. (iii) The nitrogen-carbon bond is accumulating electronic density for Y-polarization, but largely depleting the density for X-polarization.

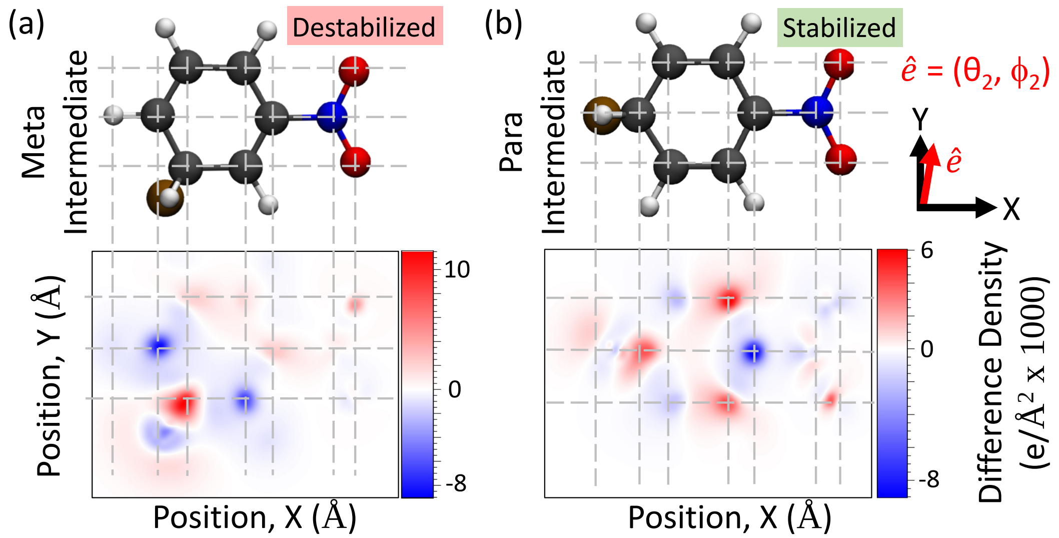

Figure S3: Ground state density difference $\Delta\rho_{00}(x, y) = \int dz[\rho^{\mathrm{M}}_{00}(x, y, z) - \xi_{00}(x, y, z)]$ of (a) the *meta*-cationic intermediate and the *para*-cationic intermediate with a light-matter coupling strength $A_0 = 0.20$ a.u. and cavity frequency $\omega_c = 1.8$ eV. The cavity polarization is along $\hat{e} = (\theta_2, \phi_2)$ defined in Fig. 1 in the main text with inset to panel (b) showing the projection of the polarization onto the XY-plane. The color bar indicates the magnitude and sign of the difference density $\Delta\rho_{00}(x, y)$, where positive (red) indicates electron charge accumulation and negative (blue) indicates electron charge depletion upon coupling the molecule with the cavity. Note that the color bar scales in panel (a) and panel (b) are different.

Fig. S3 shows the density difference between the molecule coupled inside the cavity and outside the cavity, for the *meta*-substituted (panel a) and *para*-substituted (panel b) intermediate. The cavity polarization is along $\hat{\mathbf{e}} = (\theta_2, \phi_2)$ and the inset to Fig. S3b for the polarization direction projected onto the XY-plane). The cavity parameters are the same as for Fig. 2 of the main text: $A_0 = 0.3$ a.u. and $\omega_c = 1.8$ eV. Under these conditions, the *para*-substituted Wheland intermediate becomes more stable than the *meta*-substituted intermediate by ~ 1.97 kcal/mol. Fig. S3a shows the density difference for the *meta*-substituted reaction intermediate while Fig. S3b presents the density difference for the *para*-substituted reaction intermediate. The color scheme of this plot is such that red (positive values) indicates the accumulation of electron density and blue (negative values) indicates depletion of electron density upon insertion of the chemical species into the cavity.

The primary features of the difference density contour maps are that of the localization of the electron density accumulation (red) and depletion (blue). For the *meta*-substituted

intermediate species, the choice of cavity polarization has a minimal effect. In both cases, the bromine-connected carbon accumulates a large amount of electron density while the adjacent carbons (*ortho* and *para* carbons relative to the nitro group) exhibit strong electron density depletion. The *ortho* and *para* intermediate species (Fig. 2b in the main text and Fig. S3b) instead show a delocalized electron density accumulation on the bromine-connected carbon and the two carbons in the *meta* position relative to the bromine-connected carbon. However, the electron density becomes depleted only at the *para* carbon relative to the bromine-connected carbon. This reorganization of the electron density allows for the cavity-mediated selectivity of the three cationic intermediate species.

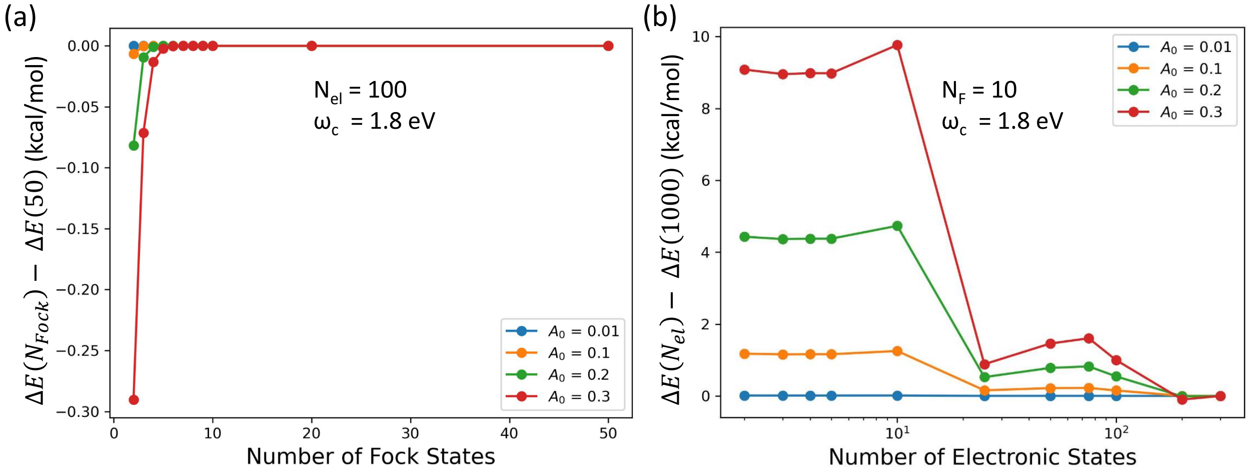

Figure S4: The convergence of the relative energy difference, $\Delta E = E_0^{\text{Para}}(\mathbf{R}) - E_0^{\text{Meta}}(\mathbf{R})$, between the *ortho* and *para* intermediate species as functions of the number of the (a) Fock and (b) electronic basis states. The colors correspond to the light-matter coupling strength A_0. In both panels, the reference energy is the case with the most basis states, *i.e.*, $N_{\text{Fock}} = 50$ and $N_{\text{el}} = 300$, respectively. In both panels, the cavity frequency is set to $\omega_c = 1.8$ eV.

Convergence of the Calculation with Adiabatic-Fock Basis

Fig. S4 provides the convergence test of the polariton ground state calculations. As has been established in our previous work for the pQED method,[1] the photonic basis is relatively easy to converge in realistic, *ab initio* systems. In this case, only 3 Fock states are required to converge below 1 kcal/mol, which is usually accepted as chemical accuracy. However, the size of the electronic basis could affect the resulting energy difference due to the dipole self-energy term which mixes, in principle, all electronic states according to the square of the dipole matrix in the truncated subspace,[1,8] $(\hat{\mathcal{P}}\hat{\mu}\hat{\mathcal{P}})^2$, where $\hat{\mathcal{P}} = \sum_{\alpha}^{N_{\text{el}}} |\psi_\alpha\rangle\langle\psi_\alpha|$.

The typical trend of the convergence will be achieved with about ~ 25 electronic basis states, and with $\sim 10^2$ electronic basis states the energy convergence will approach around 1 kcal/mol. Further, in small molecules, such as nitrobenzene, the number of excited states that LR-TDDFT can accurately resolve – or that are physical at all – may not be more than ~ 100. Our previous benchmark calculations suggest that the accuracy of pQED simulation[1] is similar to the QED-coupled cluster level of theory.[6]

Additional Analysis of the Ground State Density

For the *ortho*/*meta* intermediate species with cavity polarization along (θ_1, ϕ_1), Fig. S5/Fig. S6 shows various matrix elements of the reduced electronic density matrix $\hat{\rho}_{00}^{\mathrm{M}} = \mathrm{Tr}_{\mathrm{ph}}[\hat{\rho}_{00}] = \mathrm{Tr}_{\mathrm{ph}}[|\Phi_0\rangle\langle\Phi_0|]$ for the polaritonic ground state $|\Phi_0\rangle$ as well as matrix elements of the dipole $\hat{\mu}$ and squared dipole $\hat{\mu}^2$ operators. The contributions from the diagonal electronic state density $\xi_{\alpha\alpha}$ (Fig. S5a) and ground-to-excited electronic transition density $\xi_{0\alpha}$ (Fig. S5b) matrix elements are shown as a function of the light-matter coupling strength A_0. Note that only the significant contributions are shown for visual clarity. The ground-to-excited dipole $\mu_{0\alpha}$ (black circles) and squared dipole $(\hat{\mu}^2)_{0\alpha}$ (black stars) matrix elements in the direction of (θ_1, ϕ_1) are shown in panels (c) and (d). Strong correlations between the transition moments (black symbols) and the diagonal density and transition density elements (green triangles) can be seen which gives insights into how and which electronic excited states mix into the ground polaritonic state.

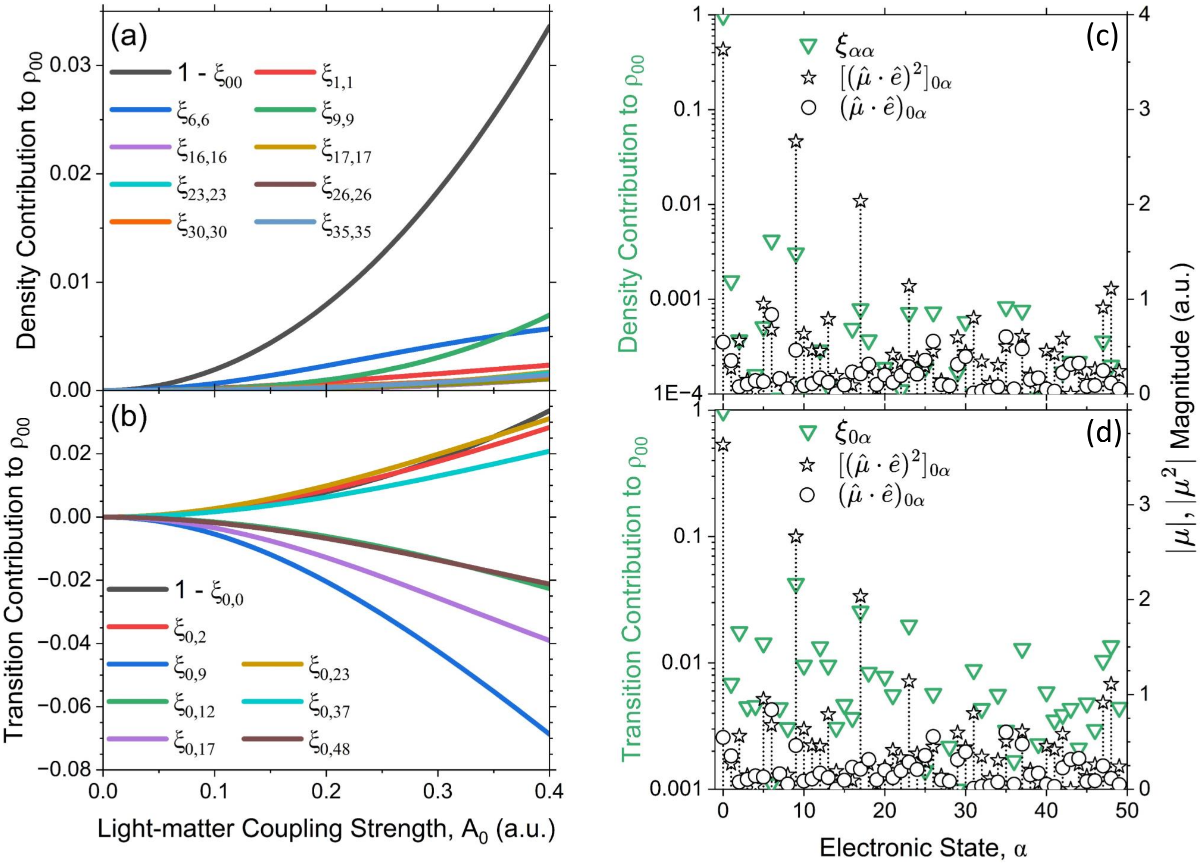

Figure S5: Contributions from various elements of the reduced electronic density matrix for the polaritonic ground state, $\hat{\rho}_{00}^{\mathrm{M}} = \mathrm{Tr}_{\mathrm{ph}}[\hat{\rho}_{00}] = \mathrm{Tr}_{\mathrm{ph}}[|\Phi_0\rangle\langle\Phi_0|]$, where $|\Phi_0\rangle$ is the polaritonic ground state defined in Eq. 4 in the main text, for the *ortho* intermediate species with cavity polarization along (θ_1, ϕ_1) (see main text Fig. 2) and cavity frequency $\omega_{\mathrm{c}} = 1.8$ eV. Contributions to the ground polaritonic density from the (a) diagonal $\xi_{\alpha\alpha}$ and (b) ground-to-excited electronic density contributions $\xi_{0\alpha}$ as functions of the light-matter coupling strength A_0. For fixed light-matter coupling strength $A_0 = 0.3$ a.u., the (c) diagonal and (d) ground-to-excited electronic density contributions (green triangles), transition dipole matrix elements (black circles), and dipole squared matrix elements (black stars). In both cases of dipole, all matrix elements were projected along the polarization direction. Note that for panels (a) and (b), only contributions above (a) 0.001 and (b) 0.01 are shown for visual clarity.

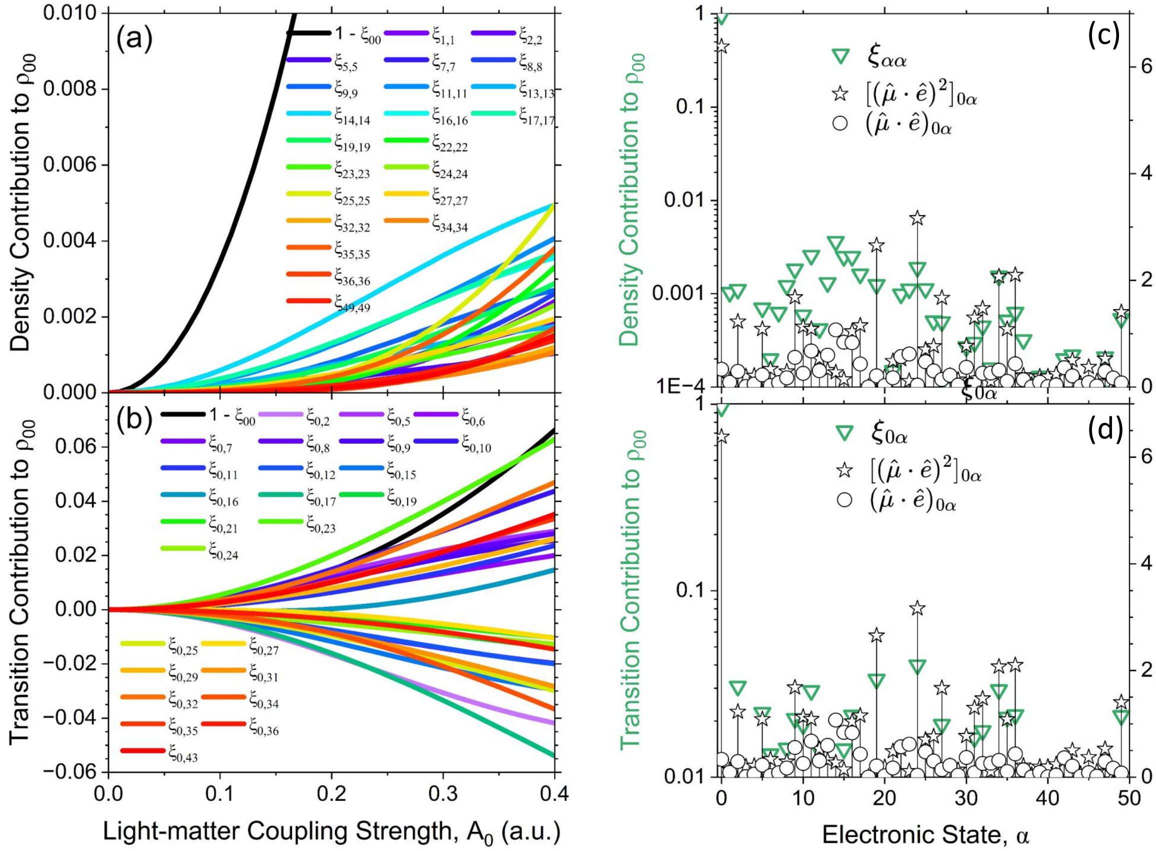

Figure S6: Contributions from various elements of the reduced electronic density matrix for the polaritonic ground state, $\hat{\rho}_{00}^{\mathrm{M}} = \mathrm{Tr}_{\mathrm{ph}}[\hat{\rho}_{00}] = \mathrm{Tr}_{\mathrm{ph}}[|\Phi_0\rangle\langle\Phi_0|]$, where $|\Phi_0\rangle$ is the polaritonic ground state defined in Eq. 4 in the main text, for the *meta* intermediate species with cavity polarization along (θ_1, ϕ_1) (see main text Fig. 2) and cavity frequency $\omega_{\mathrm{c}} = 1.8$ eV. Contributions to the ground polaritonic density from the (a) diagonal $\xi_{\alpha\alpha}$ and (b) ground-to-excited matrix elements $\xi_{0\alpha}$ of the electronic density contributions as functions of the light-matter coupling strength A_0. For fixed light-matter coupling strength $A_0 = 0.3$ a.u., the (c) diagonal and (d) ground-to-excited electronic density contributions (green triangles), transition dipole matrix elements (black circles), and dipole squared matrix elements (black stars). In both cases of dipole, all matrix elements were projected along the polarization direction. Note that for panels (a) and (b), only contributions above (a) 0.001 and (b) 0.01 are shown for visual clarity.

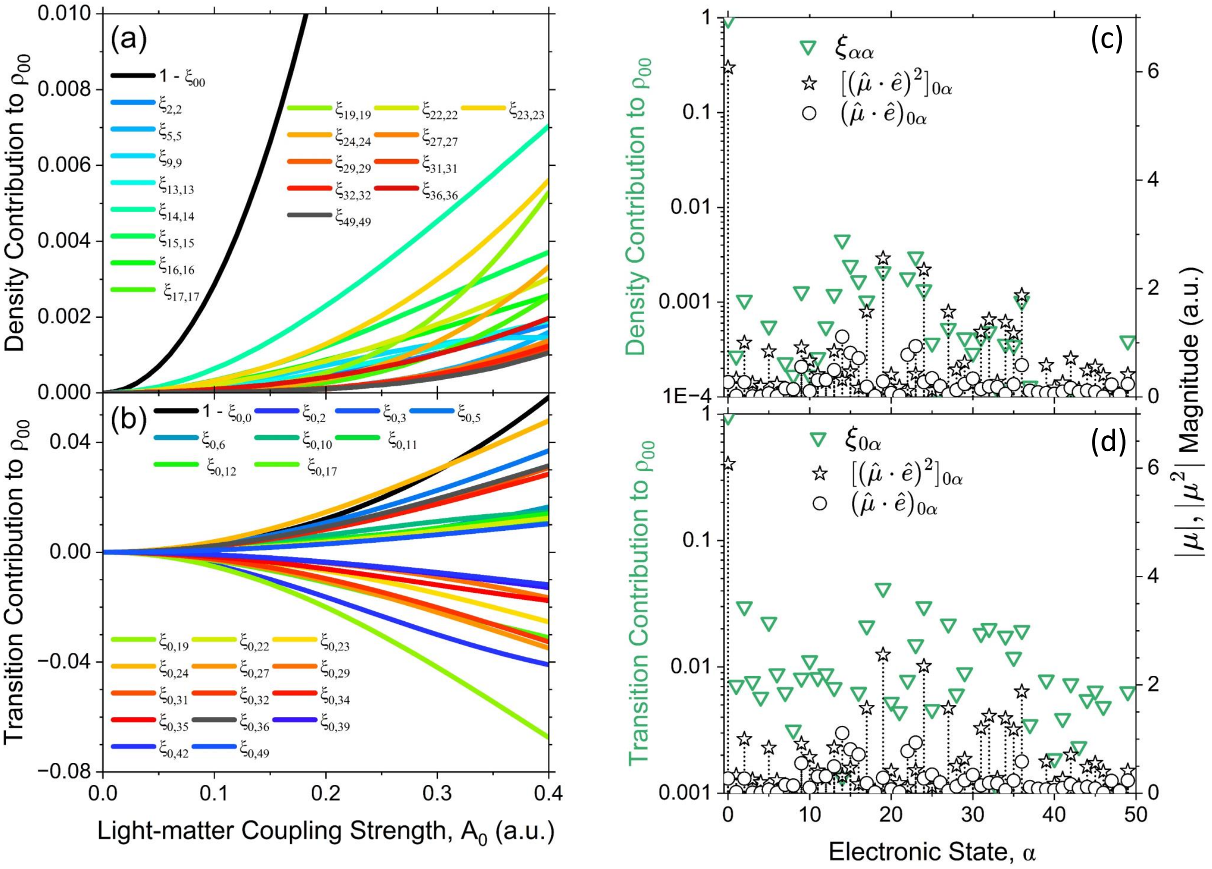

Figure S7: Contributions from various elements of the reduced electronic density matrix for the polaritonic ground state, $\hat{\rho}_{00}^{\mathrm{M}} = \mathrm{Tr}_{\mathrm{ph}}[\hat{\rho}_{00}] = \mathrm{Tr}_{\mathrm{ph}}[|\Phi_0\rangle\langle\Phi_0|]$, where $|\Phi_0\rangle$ is the polaritonic ground state defined in Eq. 4 in the main text, for the *meta* intermediate species with cavity polarization along (θ_2, ϕ_2) (see main text Fig. 2) and cavity frequency $\omega_{\mathrm{c}} = 1.8$ eV. (a) Diagonal $\xi_{\alpha\alpha}$ and (b) ground-to-excited $\xi_{0\alpha}$ electronic density contributions as functions of the light-matter coupling strength A_0. For fixed light-matter coupling strength $A_0 = 0.3$ a.u., the (c) diagonal and (d) ground-to-excited electronic density contributions (green triangles), transition dipole matrix elements (black circles), and dipole squared matrix elements (black stars). In both cases of dipole, all matrix elements were projected along the polarization direction. Note that for panels (a) and (b) only contributions above (a) 0.001 and (b) 0.01 are shown for visual clarity.

For the *meta*/*para* intermediate species with cavity polarization along (θ_2, ϕ_2), Fig. S7/ Fig. S8 shows various matrix elements of the reduced density matrix $\hat{\rho}_{00}^{\mathrm{M}} = \mathrm{Tr}_{\mathrm{ph}}[\hat{\rho}_{00}] = \mathrm{Tr}_{\mathrm{ph}}[|\Phi_0\rangle\langle\Phi_0|]$ for the polaritonic ground state $|\Phi_0\rangle$ as well as matrix elements of the dipole $\hat{\mu}$ and squared dipole $\hat{\mu}^2$ operators. The diagonal state density $\xi_{\alpha\alpha}$ (Fig. S7a) and ground-to-excited transition density $\xi_{0\alpha}$ (Fig. S7b) matrix elements are shown as a function of the light-matter coupling strength A_0. Note that only the largest contributions are shown for

visual clarity. The ground-to-excited dipole $\mu_{0\alpha}$ (black circles) and squared dipole $(\hat{\mu}^2)_{0\alpha}$ (black stars) matrix elements in the direction of (θ_1, ϕ_1) are shown in panels (c) and (d). Strong correlations between the transition moments (black symbols) and the diagonal density and transition density elements (green triangles) can be seen which gives insights into how and which electronic excited states mix into the ground state.

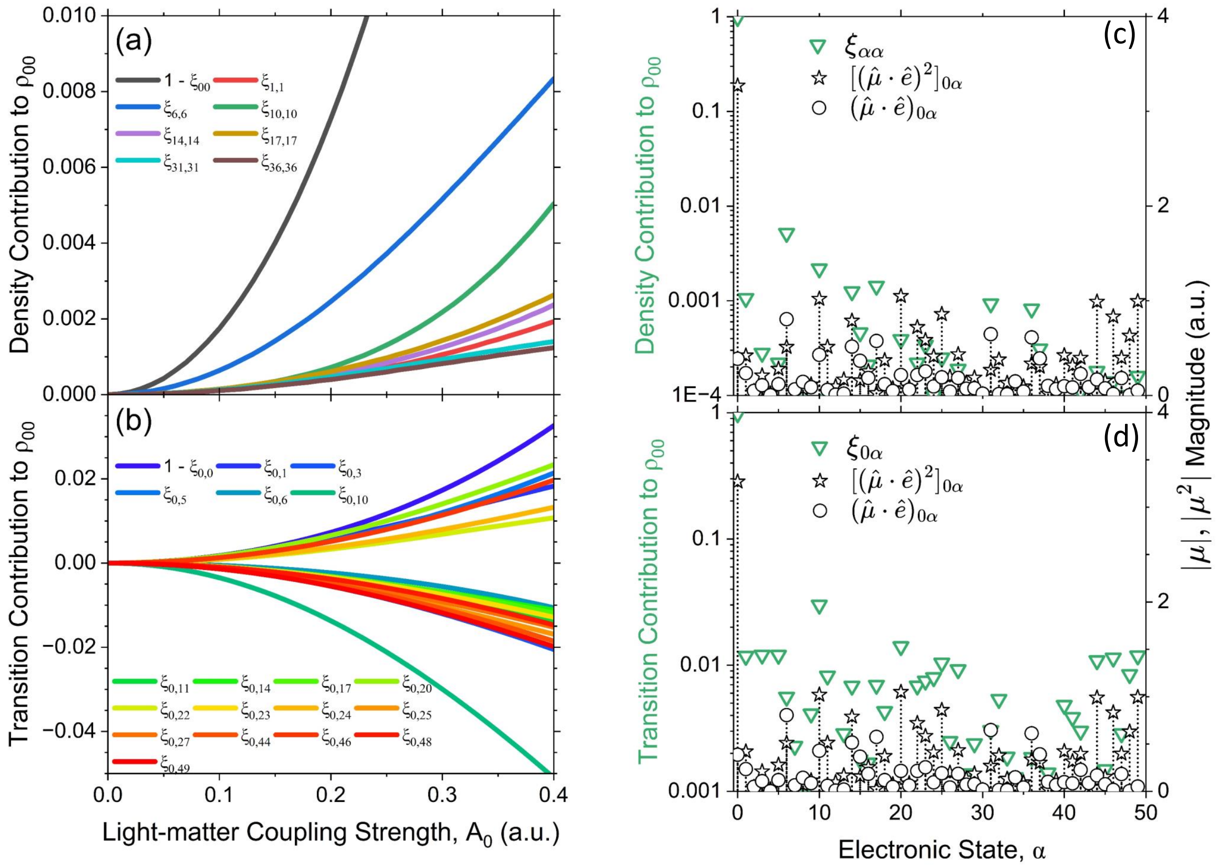

Figure S8: Contributions from various elements of the reduced electronic density matrix for the polaritonic ground state, $\hat{\rho}_{00}^{\mathrm{M}} = \mathrm{Tr}_{\mathrm{ph}}[\hat{\rho}_{00}] = \mathrm{Tr}_{\mathrm{ph}}[|\Phi_0\rangle\langle\Phi_0|]$, where $|\Phi_0\rangle$ is the polaritonic ground state defined in Eq. 4 in the main text, for the *para* intermediate species with cavity polarization along (θ_2, ϕ_2) (see main text Fig. 2) and cavity frequency $\omega_{\mathrm{c}} = 1.8$ eV. (a) Diagonal $\xi_{\alpha\alpha}$ and (b) ground-to-excited $\xi_{0\alpha}$ electronic density contributions as functions of the light-matter coupling strength A_0. For fixed light-matter coupling strength $A_0 = 0.3$ a.u., the (c) diagonal and (d) ground-to-excited electronic density contributions (green triangles), transition dipole matrix elements (black circles), and dipole squared matrix elements (black stars). In both cases of dipole, all matrix elements were projected along the polarization direction. Note: For panels (a,b) only contributions above (a) 0.001 and (b) 0.01 are shown for visual clarity.

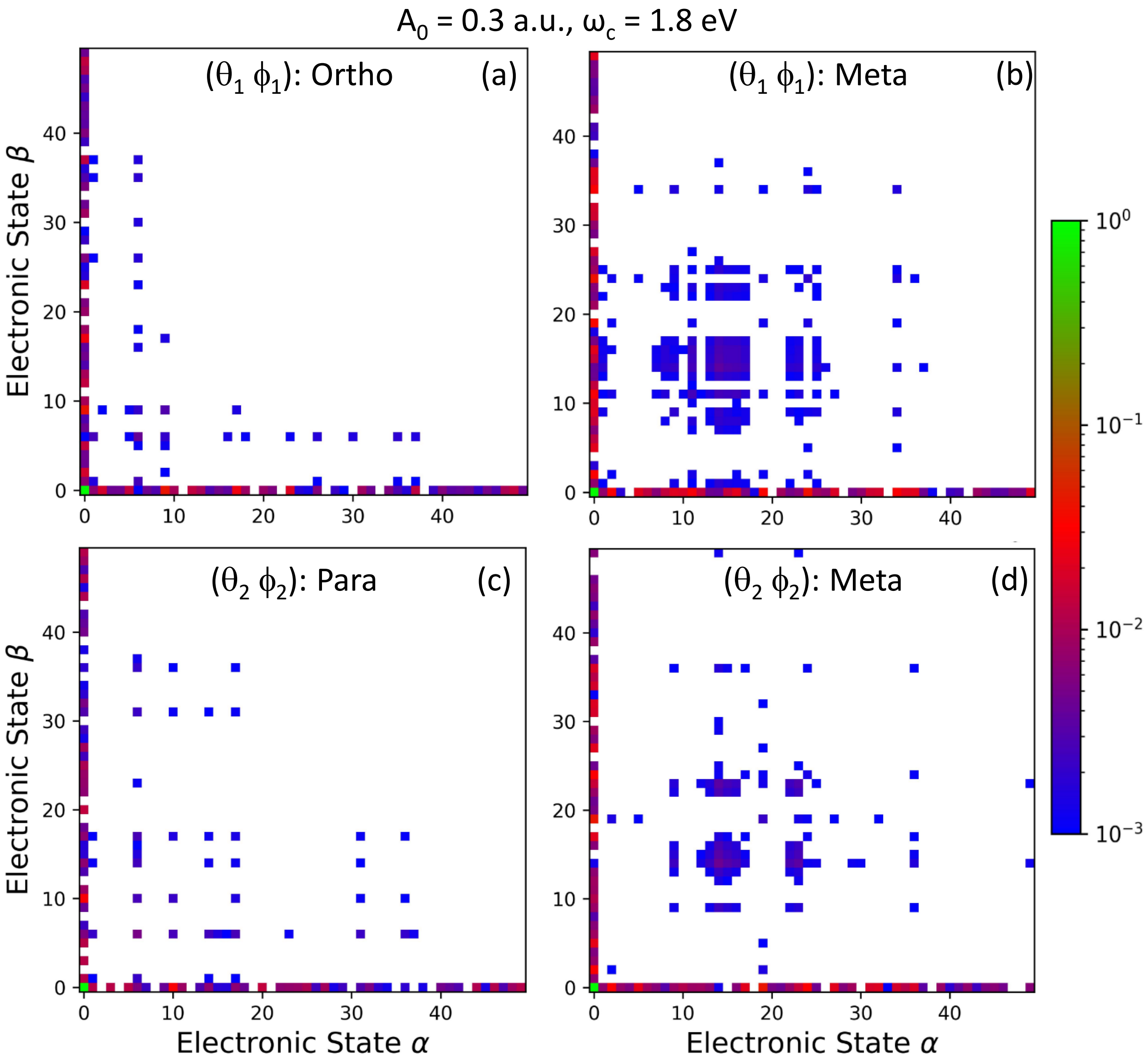

Figure S9: Contributions from various elements of the reduced density matrix, $\hat{\rho}_{00}^{\mathrm{M}} = \mathrm{Tr}_{\mathrm{ph}}[\hat{\rho}_{00}] = \mathrm{Tr}_{\mathrm{ph}}[|\Phi_0\rangle\langle\Phi_0|]$, where $|\Phi_0\rangle$ is the polaritonic ground state defined in Eq. 4 in the main text, for the (a) *ortho* intermediate species with cavity polarization along (θ_1, ϕ_1), (b) *meta* intermediate with cavity polarization along (θ_1, ϕ_1), (c) *para* intermediate species with cavity polarization along (θ_2, ϕ_2), and (d) *meta* intermediate with cavity polarization along (θ_2, ϕ_2). In all cases, the cavity frequency $\omega_{\mathrm{c}} = 1.8$ eV and the light-matter coupling strength $A_0 = 0.30$ a.u. The colorbar indicates the magnitude of the contribution from the $\alpha\beta_{\mathrm{th}}$ reduced electronic density matrix element on a log-scale.

Fig. S9 presents the reduced polaritonic ground state density matrix $\hat{\rho}_{00}^{\mathrm{M}} = \mathrm{Tr}_{\mathrm{ph}}[\hat{\rho}_{00}] = \mathrm{Tr}_{\mathrm{ph}}[|\Phi_0\rangle\langle\Phi_0|]$ for the polaritonic ground state $|\Phi_0\rangle$. The matrix elements $\langle\alpha|\hat{\rho}_{00}^{\mathrm{M}}|\beta\rangle$ are presented, with its magnitude $|\langle\alpha|\hat{\rho}_{00}^{\mathrm{M}}|\beta\rangle|$ indicated by the color bar. Four intermediate species in their respective cavity polarization directions are presented, with (a) *ortho* intermediate

species with cavity polarization along (θ_1, ϕ_1), (b) *meta* intermediate with cavity polarization along (θ_1, ϕ_1), (c) *para* intermediate species with cavity polarization along (θ_2, ϕ_2), and (d) *meta* intermediate with cavity polarization along (θ_2, ϕ_2). All panels have cavity frequency $\omega_\mathrm{c} = 1.8$ eV and light-matter coupling strength $A_0 = 0.30$ a.u. The colorbar indicates the magnitude of the contribution of each element on a log-scale.

Aside from the ground state electronic density, $\alpha\beta = 00$ (at $A_0 = 0.0$ a.u., this element is 1.0), the next largest contributions to the polaritonic ground state stem from the ground-to-excited electronic transition density, 0β and $\alpha 0$. This can be easily rationalized since the ground state expansion coefficient $C_{0,0}$ is always the largest in magnitude, and the ground-to-excited reduced electronic density matrix elements are $\sum_n C_{0n} C_{\alpha n}$. Excited-to-excited elements as well as diagonal elements are expected to be an order of magnitude smaller in magnitude following the same reasoning, since neither of those types of elements contain the ground state electronic state expansion coefficient C_{0n}. In both cases of polarization, the *meta* intermediate species (Fig. S9b,d) exhibits a larger amount of excited-to-excited density matrix elements than both the *ortho* (Fig. S9a) and *para* (Fig. S9) intermediate species.

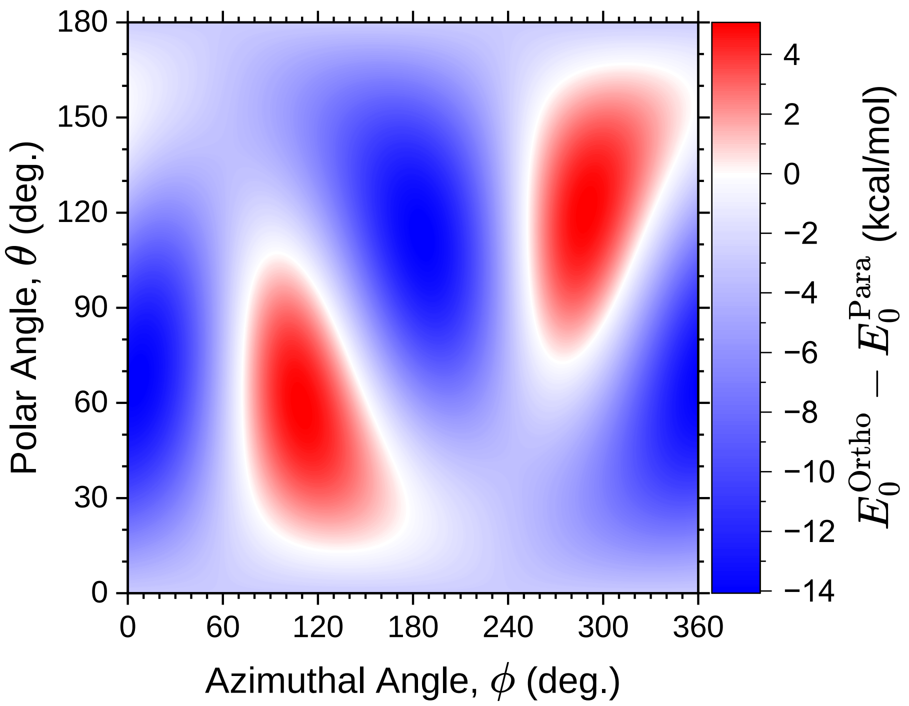

Figure S10: Relative energy of the polaritonic ground states between *ortho*-$PhNO_2$-Br^+ and *para*-$PhNO_2$-Br^+ as a function of the azimuthal $\phi \in [0, 2\pi)$ and polar $\theta \in [0, \pi)$ angles of the cavity polarization vector with respect to the molecular cartesian axes in the inset of panel (b) of Fig. 1 in the main text. The cavity frequency and coupling strength are fixed at $\omega_c = 1.8$ eV and $A_0 = 0.3$ a.u. (corresponding to a cavity volume of $\mathcal{V} = 0.15$ nm^3 or a field strength of $\mathcal{E} = 10.8$ V/nm). Blue regions indicate the angles which stabilize the *Ortho* species while red shows the stable *Para* species.

Complementary to Fig. 1 in the main text, we show the ground state energy difference between the *ortho* and *para* intermediate species as a function of the cavity polarization direction (ϕ, θ). These results were computed using the same cavity parameters as that of Fig. 1 in the main text: light-matter coupling strength $A_0 = 0.3$ a.u. and cavity frequency $\omega_c = 1.8$ eV. This energy difference showcases the relative kinetics between the two products that cannot be observed outside the cavity.

References

(1) Weight, B. M.; Krauss, T. D.; Huo, P. Investigating Molecular Exciton Polaritons Using Ab Initio Cavity Quantum Electrodynamics. *J. Phys. Chem. Lett.* **2023**, *14*, 5901–5913.

(2) Epifanovsky, E. et al. Software for the frontiers of quantum chemistry: An overview of developments in the Q-Chem 5 package. *J. Chem. Phys.* **2021**, *155*, 084801.

(3) Furche, F.; Ahlrichs, R. Adiabatic time-dependent density functional methods for excited state properties. *J. Chem. Phys.* **2002**, *117*, 7433–7447.

(4) Furche, F.; Ahlrichs, R. Adiabatic time-dependent density functional methods for excited state properties. *The Journal of Chemical Physics* **2002**, *117*, 7433–7447.

(5) Tretiak, S.; Chernyak, V. Resonant nonlinear polarizabilities in the time-dependent density functional theory. *The Journal of Chemical Physics* **2003**, *119*, 8809–8823.

(6) Pavošević, F.; Hammes-Schiffer, S.; Rubio, A.; Flick, J. Cavity-Modulated Proton Transfer Reactions. *J. Am. Chem. Soc.* **2022**, *144*, 4995–5002.

(7) Pavošević, F.; Smith, R. L.; Rubio, A. Computational study on the catalytic control of endo/exo Diels-Alder reactions by cavity quantum vacuum fluctuations. *Nat Commun* **2023**, *14*, 2766.

(8) Mandal, A.; Taylor, M. A. D.; Weight, B. M.; Koessler, E. R.; Li, X.; Huo, P. Theoretical Advances in Polariton Chemistry and Molecular Cavity Quantum Electrodynamics. **2023**, *123*, 9786–9879.
